# Machine learning-driven optimization of alkali-activated sustainable pavement concrete incorporating agro-industrial wastes

**DOI:** 10.1038/s41598-026-55050-6

**Published:** 2026-06-08

**Authors:** Akhila Sheshadri, Shriram Marathe, Łukasz Sadowski, Bhagya Shree

**Affiliations:** 1 Department of Civil Engineering, Nitte (Deemed to be University), NMAM Institute of Technology (NMAMIT), Nitte, Karkala, Udupi District, Karnataka 574110 India; 2 Department of Materials Engineering and Construction Processes, Faculty of Civil Engineering, Wroclaw University of Science and Technology, PolitechnikaWroclawska 27, 50-370 Wrocław, Poland; 3 Manipal Institute of Technology, Manipal Academy of Higher Education, Manipal, 576104 India

**Keywords:** Alkali activated concrete, Machine learning, Random forest, Support vector machine, Gradient boosting, Sustainable construction, Engineering, Environmental sciences, Materials science

## Abstract

The integration of agro-industrial wastes into alkali-activated concrete (AAC) offers a sustainable alternative to traditional concrete pavement material, yet the synergistic effects of waste foundry sand (WFS) as a fine aggregate replacement and rice husk ash (RHA) as a partial binder replacement in ground granulated blast furnace slag (GGBS)-based AAC designed for rigid pavement applications remain under-explored. This study investigates the machine learning (ML)-driven optimization of AAC, addressing a critical research gap in material synergy and predictive modeling. Experimental results indicate that increasing WFS (0–30%) and RHA (0–20%) reduces compressive strength (CS) to some extent; however, an optimized mix containing 20% WFS and 15% RHA achieved a 28-day CS satisfying pavement-quality concrete requirements while maintaining workability within acceptable limits. A comparative ML framework comprising six algorithms; Multiple Linear Regression, Decision Tree, Random Forest, AdaBoost, Support Vector Regression, and Gradient Boosting was developed and benchmarked to predict the CS of 330 experimental samples. The Random Forest model achieved the highest predictive accuracy (R^2^ = 0.9176, RMSE = 2.99 MPa, MAE = 2.62 MPa), with performance statistically comparable to Gradient Boosting and AdaBoost. Feature importance and interpretability analysis revealed that GGBS and fine aggregate content significantly influence CS, while higher WFS and RHA levels adversely affect strength. This research provides a scalable framework for designing low-carbon pavement materials by bridging the gap between experimental mix design and advanced computational optimization that substantially reduce dependence on resource-intensive experimental trials for AAC incorporating agro-industrial wastes

## Introduction

The construction sector is responsible for approximately 23% of global CO₂ emissions linked to worldwide economic activities^1^. Over recent decades, the growing demand for cement in the construction industry has led to the accelerated extraction of non-renewable resources^2–4^. To mitigate these environmental impacts, researchers have investigated resource-saving techniques and ways to minimize the ecological footprint of concrete production^5^. One promising approach is utilizing industrial by-products to reduce the environmental burden of ordinary portland cement concrete (OPCC)^6^. Alkali-activated materials have emerged as essential alternatives for developing low-CO₂ cement and concrete^6^ with alkali-activated concrete (AAC) demonstrating competitive mechanical performance and improved durability relative to OPCC under several exposure conditions^7–9^. AAC is gaining popularity as a sustainable, durable structural material^10^, and research underscores its potential to reduce carbon emissions and promote effective waste management by replacing cement with binders derived from industrial by-products^10,11^.

Rice is widely cultivated across Asia and other continents, with global paddy production reaching approximately 769 million tonnes in 2017^12^ Rice husk combustion in cogeneration plants produces about 20% of its mass as rice husk ash (RHA)^13,14^. Although RHA has multiple commercial applications, large quantities are often discarded in landfills. In India, for instancethe world’s second-largest rice producerless than 1% of RHA is productively utilized, leaving millions of metric tons to accumulate annually^15,16^. The uncontrolled disposal and burning of rice husk residues contribute substantially to particulate pollution and greenhouse gas emissions^17^, reinforcing the urgency of valorizing RHA as a construction material. Research has demonstrated that partially replacing ground granulated blast furnace slag (GGBS) with RHA in AAC yields satisfactory compressive strength (CS) and reduces alkali-silica reaction expansion^18^. A review by Anju et al. further highlights the potential of alkali-activated systems incorporating RHA when activated with sodium hydroxide and sodium silicate solutions^19^.

Highway pavement construction demands large quantities of fresh materials, with mineral aggregates constituting over 80% of concrete pavement volume by mass. The twin pressures of increasing industrial waste generation and diminishing natural aggregate reserves underscore the need for efficient recycling and reuse strategies^20^. Waste foundry sand (WFS), a by-product of the metal casting industry, is commonly discarded in landfills^20^. Recent studies, have investigated its potential in construction materials to alleviate disposal issues and foster sustainability^21^. Studies have shown that incorporating treated WFS in slag-based geopolymer mortars can significantly enhance their compressive strength, adhesion, and flexural properties while maintaining environmental safety within regulatory limits^22^. WFS also shows promise as a replacement for natural fine aggregates in conventional concrete, effectively reducing the demand for virgin river sand^21^. The broader strategy of combining industrial and agricultural wastes as complementary concrete ingredients has gained traction in recent literature; Padavala et al.^23^ demonstrated the feasibility of using fly ash (an industrial waste) as a supplementary binder alongside coconut shell (an agricultural by-product) as a coarse aggregate replacement, with ANN-based strength prediction confirming the viability of this dual-waste approach for structural concrete. Moreover, ternary geopolymer mixes incorporating combinations of FA, GGBS, and RHA, along with natural or foundry sands, have been investigated to achieve high mechanical performance and environmental benefits. Comparative analyses by Choudhary et al.^24^ report that blends containing FA, GGBS, and Metakaolin exhibit superior mechanical properties compared to those with RHA. However, the latter still meets the required strength criteria and offers a viable alternative in specific applications. In the context of pavement infrastructure specifically, Paluri et al.^25^ demonstrated that geopolymer paver blocks synthesized with GGBS, fly ash, and reclaimed asphalt pavement aggregates satisfied the mechanical and durability requirements of IS 15,658:2021, confirming the suitability of GGBS-based alkali-activated systems for pavement applications while significantly reducing the carbon footprint relative to conventional cement-based paver blocks.

The integration of machine learning (ML) into concrete research has gained substantial momentum, particularly for predicting 28-day compressive strength from mix composition data a task that traditionally requires extensive and time-consuming experimental campaigns^26,27^. Various studies have demonstrated the effectiveness of ensemble and hybrid ML approaches in capturing complex nonlinear relationships between input variables and mechanical performance. For instance, hybrid optimized Boost models have been successfully employed for compressive strength prediction of cement-based materials, highlighting the potential of advanced boosting algorithms in enhancing prediction accuracy and robustness^28^. Similarly, recent work integrating ML with interpretability techniques such as Shapley Additive Explanations (SHAP) has provided deeper insights into feature contributions and model behavior in sustainable concrete systems^29^. ML-based predictive models not only improve the accuracy of strength forecasts but also reduce reliance on laboratory trials, offering significant practical advantages in mix design optimization^30^. Among classical algorithms, backpropagation neural networks (BPNN), artificial neural networks (ANN), and support vector regression (SVR) have been widely applied; for example, Vijay and Murmu^31^ demonstrated satisfactory BPNN predictions for concrete containing recycled aggregates, and Kadhum^32^ successfully predicted the tensile strength of reactive powder concrete using ANN–SVR hybrid models. Ensemble learning methods have further elevated predictive performance: Karthiyaini et al.^33^ applied hybrid AI approaches to fibre-reinforced concrete with improved accuracy over single-model methods, while Huang et al.^34^ combined the Firefly Algorithm with Random Forest (RF) to forecast the compressive strength of concrete incorporating alternative aggregates, reporting high predictive fidelity. Gradient Boosting Regression (GBR) has similarly demonstrated superior performance to SVR for tensile strength prediction^35^, and ensemble models particularly RF and AdaBoost have shown robustness in capturing the nonlinear, multicollinear relationships characteristic of multi-component concrete systems^36^. In addition to predictive modeling, ML techniques have also been applied in broader civil engineering domains, including structural health monitoring and damage detection. For example, ML-assisted acoustic emission analysis has been utilized for localization of wire-break events in prestressed structural elements, demonstrating the versatility and applicability of data-driven approaches in complex engineering problems. These developments underline the growing importance of ML in both material design and structural performance assessment^37^.

In the specific context of concrete incorporating both RHA and WFS, several ML studies have been published in recent years. Wang et al.^38^ employed M5P model trees, gene expression programming, ANN, and RF to predict the compressive strength of OPC-based concrete containing ternary blend, finding that RF yielded the most accurate forecasts. Abdalla et al.^39^ investigated similar OPC-based concrete using Multi-Layer Perceptron (MLP) networks and confirmed high prediction accuracy. Huang et al.^40^ applied a Firefly–RF hybrid to concrete with alternative aggregates, achieving accurate CS predictions. Abdulrahman et al. assessed ensemble and individual models for concrete binders made with RHA and WFS, concluding that a DT-AdaBoost model and an enhanced bagging approach delivered the best predictive performance. Taken together, these studies establish that RF and ensemble methods are effective for predicting CS in concrete systems containing RHA and WFS. However, all four studies were conducted on Ordinary PortlandCement (OPC)-based or conventional geopolymer systems not on AAC with GGBS as the sole binder and none targeted rigid pavement-quality concrete satisfying the strength requirements prescribed by Indian design codes^41^.

Although WFS and RHA have been individually studied in OPC-based concrete and, to a limited extent, in geopolymer systems, their combined binary substitution WFS as a partial fine aggregate replacement and RHA as a partial GGBS binder replacement in fully GGBS-based AAC has not been reported in the open literature. The unique activation chemistry of GGBS-based AAC (governed by Ca-Al-Si gel formation, activator modulus, and water-to-binder ratio) differs fundamentally from OPC and fly-ash-based geopolymers, making it inadmissible to extrapolate findings from those systems to GGBS-AAC without independent experimental investigation.

Existing ML studies on AACs have primarily focused on general-purpose structural applications, often employing a limited number of predictive models and providing minimal interpretability of model outputs. Furthermore, most studies consider single waste streams or conventional SCMs, with limited attention to the combined incorporation of agro-industrial wastes within a pavement-specific design framework. In particular, the applicability of such systems to rigid PQCs, which is governed by distinct performance requirements (IRC:44-2017), remains insufficiently explored. Additionally, the prior studies generally emphasize predictive accuracy without systematically examining ML model robustness across diverse algorithms or providing insight into the relative influence of input parameters. This limits the practical usability of ML models for mix design optimization in complex multi-component systems such as GGBS-based AACs incorporating both RHA and WFS.

To address these gaps, the present study makes 3 key contributions. First, it experimentally evaluates the combined influence of WFS (0–30%) and RHA (0–20%) on the fresh and hardened properties of GGBS-based alkali-activated concrete specifically designed for pavement applications. Second, it develops and systematically benchmarks 6 ML models (MLR, DT, RF, SVR, AdaBoost, and GBR) for CS prediction within this material system. Third, it integrates model interpretability analysis to identify the relative significance of constituent materials, thereby linking data-driven predictions with material behavior.

These collective contributions jointly provide a structured framework for data-driven optimization of sustainable PQC mixtures (AACs) incorporating agro-industrial wastes, while maintaining compliance with performance requirements relevant to high-traffic volume—rigid pavement applications.

## Materials

**Fig. 1:**
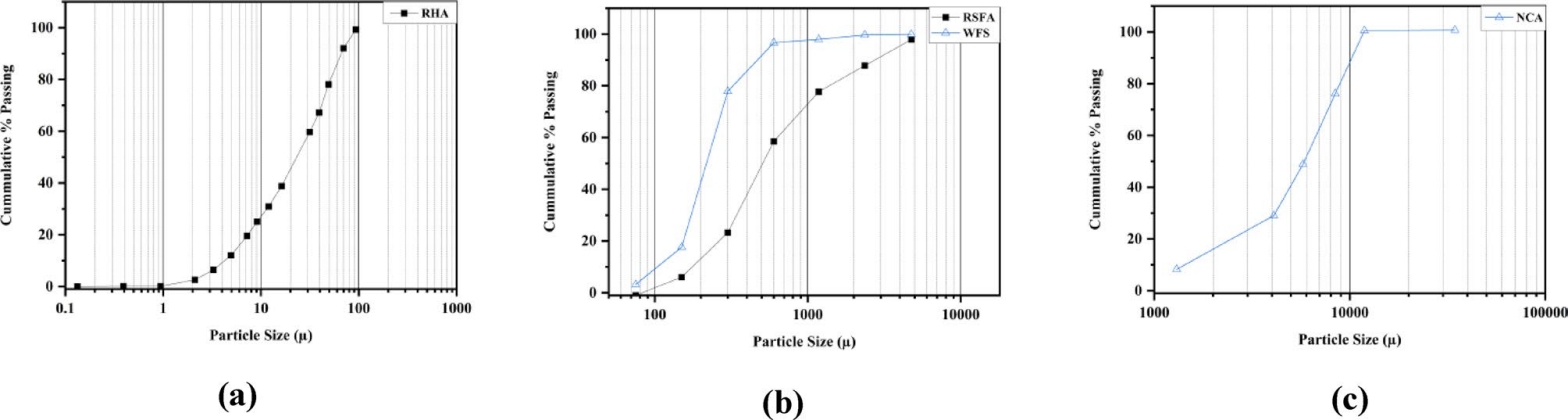
Particle size distribution of (**a**) RHA (**b**) RSFA and WFS (**c**) NCA.

Ground Granulated Blast Furnace Slag (GGBS) was supplied by JSW, Thoranagallu, India and was acquired from a local supplier. The properties of GGBS, including its chemical and physical properties, are obtained from the research work of Sheshadri^21^ and are in accordance with the necessary BIS^42^ and BS^43^ standards. To incorporate RHA in this study, the material was first dried and sieved, following the method outlined by Sheshadri^44^. The RHA was dried until it reached a consistent mass and then passed through a 1.18 mm sieve to remove contaminants. After sieving, the material was pulverized using air-milling for 5–25 min, resulting in an average particle diameter of approximately 7 µm. The chemical composition of RHA has been represented in Table 1. Natural Coarse Aggregate (NCA) was procured from local vendors. Crushed, well-graded, angular, clean granite aggregates up to the highest aggregate size of 20 mm in accordance with IRC44:2017^41^ were employed. It has the specific gravity is 2.71, the fineness modulus is 6.13, both verified by IS2386 Part-3^45^, and it has a water absorption rate of 0.28%. The River Sand Fine Aggregate(RSFA) used in this study has a specific gravity of 2.62, the fineness modulus is 2.24 conforming to IS2386 Part-3^45^ standards, and falls under zone II according to IS 383:2016^46^ and it has a water absorption of 2.11. The Waste Foundry Sand (WFS) used in this study was sourced from Lamina Foundry Industries in Nitte, Udupi District, India. The WFS had a specific gravity of 2.4 IS2386 Part-3^45^ and a fineness modulus of 3.0. Its water absorption was 3.80%, and it exhibited a unimodal particle size distribution, with the majority of the particles falling within the 150 µm to 300 µm range. As a result, the WFS does not clearly belong to any specific grading zone. The particle size distribution curves for RHA, SFA, NCA and WFS has been represented in Fig. 1. The alkaline activator solution is employed to activate the cementing property of pozzolanic binders of AAC mixes. In the current study, LSS, i.e., Na_2_SiO_3_ along with NaOH chips (98% purity) served as the alkaline activator solution. These chemicals were procured from a local chemical supplier. The composition of LSS can be described as follows: It consists of 14.70% sodium oxide (Na_2_O), 32.80% silicon dioxide (SiO_2_), and 52.50% water (H_2_O) as per IS 14212:1995^47^. The “activator modulus” (Ms) defined as the "ratio of SiO_2_ to Na_2_O" in LSS was 2.23. The dosage of NaOH in the LSS was adjusted to generate an Ms value of 1.25 when LSS + NaOH were combined with water; this ratio was maintained throughout the AAC investigations. To obtain the desired Ms of 1.25, the alkaline activator solution was made at least 24 hours prior to the concrete production by combining the NaOH chips with LSS and water. At this point, the water-to-binder (w/b) ratio was 0.20; later, while preparing the AAC concrete^48^, more water was added to raise the w/b ratio to 0.36. Laboratory tap water, devoid of contaminants and other potentially hazardous compounds, in conjunction with the BIS^49^ requirements, was used.

**Table 1:** Chemical composition of RHA.

Constituents (wt%)	RHA
Silica oxide (SiO_2_)	90.35
Aluminium oxide (Al_2_O_3_)	0.61
Calcium oxide (CaO)	0.785
Magnesium oxide (MgO)	0.445
Potassium oxide (K_2_O)	2.71
Iron oxide (Fe_2_O_3_)	0.475
Sulphur trioxide (SO_3_)	0.38
Sodium oxide (Na_2_O)	0.24
Loss of Ignition (LOI)	4.005

## Mix design

### Mix design and AAC specimen preparation

Eleven AAC mixtures were developed for this study. The initial reference mix was designed based on previous studies^50,51^ to meet high-strength pavement requirements, targeting a required workability, which corresponds to a compaction factor value (CFV) in the range of 0.75–0.92. The mix designs, including the proportions of WFS and RHA, are summarized in Tables 2 and 3. After casting, the specimens were kept at room temperature for initial setting and then cured under controlled conditions. The curing was carried out at an ambient temperature of approximately 27 ± 2 °C, with relative humidity conditions representative of laboratory environment^48^. The specimens were water cured for 7 days followed by air curing. Following demoulding, the specimens were maintained under the same ambient conditions until the testing age. The controlled curing regime was adopted to ensure uniform strength development and to minimize variability in the experimental results^52^. In the Indian context, PQC for national highways is designed for a 28-day characteristic compressive strength of 40–45 MPa, whereas high-traffic expressways, urban arterials, and runway-grade pavements typically require 45–55 MPa, with some specifications citing values up to 65 MPa for heavy-duty applications. Accordingly, the reference mix in this study was proportioned to achieve a target 28-day CS in the range of 55–65 MPa, which represents the upper tier of PQC requirements and ensures that strength reductions due to WFS and RHA incorporation are evaluated against a demanding but realistic baseline. Beyond compressive strength, rigid pavement performance is governed by a suite of mechanical and durability requirements: adequate workability for slip-form or fixed-form paving (CFV 0.86–0.92; density above 2400 kg/m^3^ to resist abrasion and traffic-induced wear; low water absorption (SWA < 5%) and low volume of permeable voids (VPV < 10%) to ensure durability against moisture ingress and a modulus of elasticity (MOE) above 30 GPa to provide adequate load-spreading capacity under wheel loads.

**Table 2:** Mix design of AAC mixes with varying percentages of WFS.

Mix ID	SA-0-0	SA-5-0	SA-10-0	SA-15-0	SA-20-0	SA-25-0	SA-30-0
% Replacement of WFS	0%	5%	10%	15%	20%	25%	30%
GGBS	493	493	493	493	493	493	493
RHA	0	0	0	0	0	0	0
NaOH	11.2	11.2	11.2	11.2	11.2	11.2	11.2
LSS	75.12	75.12	75.12	75.12	75.12	75.12	75.12
Water	138.02	138.02	138.02	138.02	138.02	138.02	138.02
CA	1071.4	1071.4	1071.4	1071.4	1071.4	1071.4	1071.4
RSFA	577.84	548.95	520.05	491.16	462.27	433.38	404.49
WFS	0	26.63	53.26	79.9	106.53	133.17	159.8

**Table 3:** Mix design of AAC mixes with varying percentages of RHA.

Mix ID	SA-20-5	SA-20-10	SA-20-15	SA-20-20
% Replacement of RHA	5%	10%	15%	20%
GGBS	468.35	443.7	419.05	394.4
RHA	24.65	49.3	73.95	98.6
NaOH	11.2	11.2	11.2	11.2
LSS	75.12	75.12	75.12	75.12
Water	138.02	138.02	138.02	138.02
CA	1071.4	1071.4	1071.4	1071.4
RSFA	462.27	462.27	462.27	462.27
WFS	106.53	106.53	106.53	106.53

This study evaluated all these parameters, making it possible to assess the suitability of each AAC mix as a holistic pavement material rather than optimizing for compressive strength alone. Hence, the reference mix in this study was proportioned to satisfy these higher performance requirements, which differ from the lower design strength ranges commonly reported in other regions^50,51^. The AAC mixes were labeled with the abbreviation SA-0–0, where “SA” denotes slag-based pavement-quality AAC mixes, and the subsequent numbers indicate the proportions of WFS and RHA replacements. For the study, 50% of coarse aggregate (CA) was used, finer than a 20 mm IS sieve and 50% finer than a 12.50 mm IS sieve. WFS was replaced at intervals of 5%, ranging from 0 to 30%, and CS tests were conducted. Based on the test results obtained, the optimal WFS replacement was determined to be 20% without compromising the design strength. After optimizing WFS content, four additional AAC mixes were developed to study the effect of varying RHA as a fractional replacement for the primary binder. GGBS was replaced in intervals of 5%, from 0 to 20%. The CS tests showed that the optimal RHA replacement percentage, was 15%.

## Methodology

This study introduces a hybrid experimental-computational methodology, which integrates rigorous experimental testing of AAC mixes with state-of-the-art machine learning techniques. Specifically, six ML modelsMultilinear Regression (MLR), Decision Tree(DT), Random Forest(RF), AdaBoost, Support Vector Regression(SVR), and Gradient Boosting(GBR)are applied to predict CS. This combination of experimental and computational approaches has not been extensively explored in prior research on AAC.

This approach reduces the reliance on extensive experimental trials, which are often labor-intensive and time-consuming, while ensuring that the predictive framework remains robust across different modeling strategies. By benchmarking several algorithms, the study identifies the most effective methods for capturing the complex, nonlinear interactions among material constituents of AAC. The integration of comparative ML analysis with experimental validation thus provides both scientific insight and practical tools for advancing sustainable pavement construction.

### Experimental tests

**Fig. 2:**
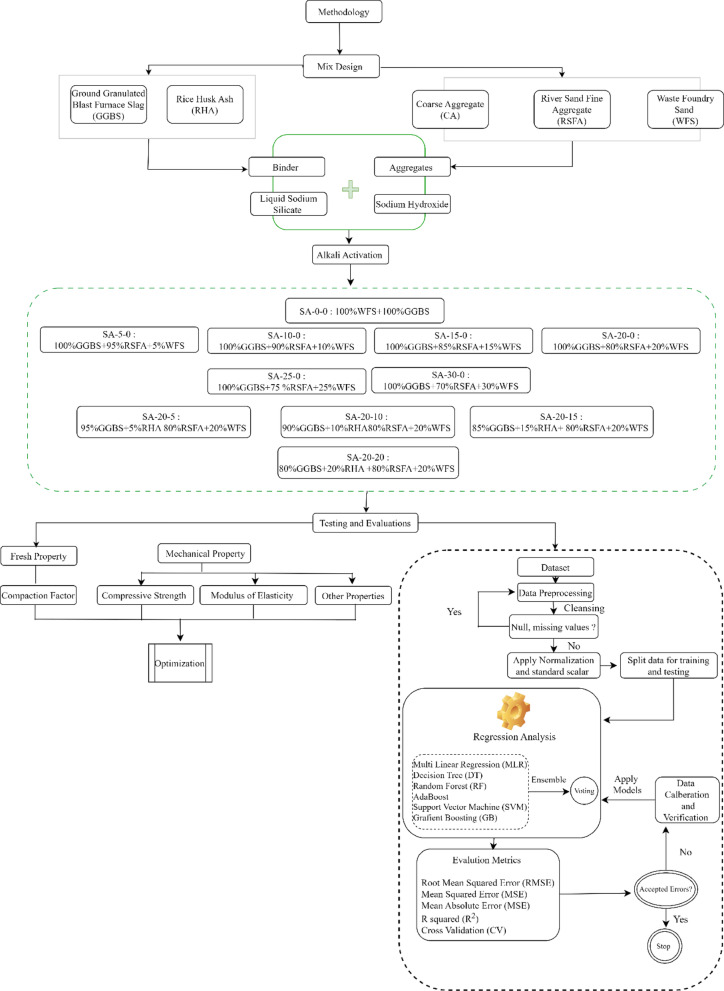
The Detailed flowchart for the experimental program and analysis.

**Fig. 3:**
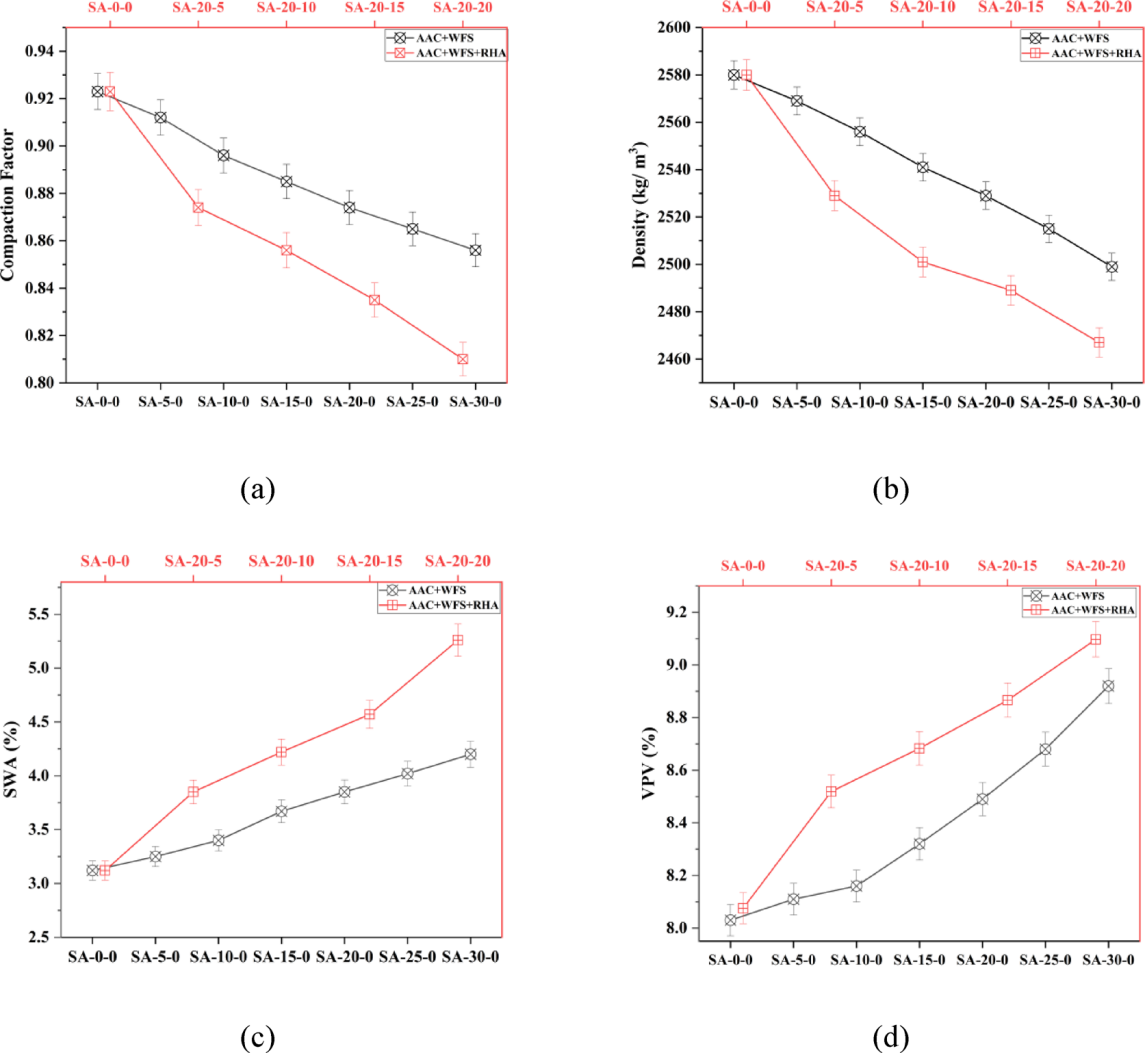
The properties of AAC mixes are as follows: (**a**) CFV with different proportions of WFS and RHA; (**b**) Variation in density with varying percentages of WFS and RHA; (**c**) SWA with different proportions of WFS and RHA; and (**d**) VPV with varying proportions of WFS and RHA.

Figure 2 illustrates the experimental and analytical framework (methodology), detailing the steps followed for casting, testing and the prediction models of the AAC mixes. Multiple tests were conducted according to the relevant standards, the details of which are as follows. For CS determination, AAC cubes (100 mm × 100 mm × 100 mm) were prepared as per IS516:2021^53^. The AAC mixes were optimized based on the CS test results, aligning with the design strength requirements. After 28 days of curing, the Volume of Permeable Voids (VPV), Saturated Water Absorption (SWA), and density of the hardened AAC mixes were measured according to ASTM C642-21^54^, with results shown in Fig. 3. Additionally, the Modulus of Elasticity (MOE) of the AAC mixes was evaluated at 28 days, as per ACI 318-2019^55^, which are represented in Fig. 3. Table 1 represents the various ML models adapted for the study and their key features.

### Data collection and description

ML is being widely applied across various domains and is continuously being refined to enhance its effectiveness^56,57^. This study utilizes a dataset of 330 samples, where 70% (231 samples) are randomly assigned as the training set, and the remaining 30% (99 samples) are used as the test set to evaluate the trained model. The goal is to predict the CS using ML techniques, including Multilinear Regression (MLR), DT, RF, AdaBoost, SVR, and GBR. The models are developed using the Sci-kit Learn library in Python. A robust and representative dataset is critical for the success of machine learning models. In this study, a total of 330 experimental samples were collected. Table 5 presents the statistical features of the variables in these samples. The input variables include GGBS, RHA, NaOH, LSS, NCA, RSFA, WFS, and Water. The dataset shows variation in the content of GGBS, RHA, RSFA and WFS across the samples. The aim of this computational modeling work is to develop metamodels using machine learning techniques that predict the CS as a mathematical function of these 8 input variables.

As mentioned, the dataset used in this study comprises 330 experimental observations obtained from 11 distinct AAC mix proportion designs, with 30 specimens tested for each mix. While no duplicate data entries exist, specimens corresponding to a given mix share identical input parameters (i.e., the portions of GGBS, RHA, RSFA, WFS, etc..), and variability arises from differences in experimentally measured CS. Thus, the dataset captures both the deterministic effect of mix design variables and the inherent variability in material response. The dataset was divided into training (70%) and testing (30%) subsets using random sampling. Although individual observations are unique, it is acknowledged that specimens from the same mix may be present in both subsets due to identical input features. To ensure robustness of the predictive framework, 10-fold cross-validation was additionally employed to evaluate model generalization. The dataset is limited to the investigated material combinations and curing conditions; therefore, the developed models are most reliable within this defined parameter space. The dataset used in this study can be made available upon reasonable request to ensure transparency and reproducibility.

### Machine learning methods

Table 4 indicates the detailed note on the adopted ML methods and their key features, provided for the brief understanding.

**Table 4:** Machine learning methods for regression and it’s key features.

Method	Description	Key Features
Multiple Linear Regression (MLR)	Extends basic linear regression to include multiple independent variables, enabling analysis of correlations between variables and predictions of continuous or categorical outputs	Relies on appropriate variable selection for reliability; valuable for understanding complex relationships and testing hypotheses
Decision Tree Regression (DT)	Utilizes hierarchical partitioning of data based on feature values to predict continuous outputs	Structured with root, decision, and leaf nodes; prone to overfitting but can be mitigated using thresholds for splits or minimum samples per leaf
Random Forest Regression (RF)	Ensemble learning method combining multiple decision trees trained on random samples and features to enhance robustness and reduce overfitting	Employs bagging and averaging for regression; handles high-dimensional data effectively; final output based on aggregated predictions of individual trees
Support Vector Regression (SVR)	Maps data into higher-dimensional space using kernel functions to model non-linear relationships; optimizes hyperplane and margins for best performance	Kernel options include linear, RBF, and polynomial; employs ε-insensitive loss to focus on critical data points; suited for both linear and non-linear problems.In the current investigation referenced, a linear kernel was employed,
AdaBoost Regression	Boosting technique combining weak learners (e.g., decision stumps) iteratively, assigning higher weights to misclassified cases to improve performance	Focuses on challenging instances through adaptive weighting; effective for capturing non-linear relationships and reducing prediction error
Gradient Boosting Regression (GBR)	Sequential ensemble learning method employing gradient descent to minimize residual errors across iterations; combines weak learners to create a robust model	Includes learning rate parameter to control contribution of trees; mitigates overfitting through random subsampling; suitable for numerical output predictions

### Hyperparameter tuning and optimization strategy

To ensure reliable predictive performance and enhance model generalizability, hyperparameter tuning and optimization were carried out for the machine learning models implemented in this study using the Scikit-learn library in Python. A hybrid optimization approach was adopted; wherein key hyperparameters were selected based on iterative model evaluation and subsequently refined using grid search techniques for selected models. This approach enabled a balance between computational efficiency and model accuracy while maintaining reproducibility. The Linear Regression model was implemented using the ordinary least squares method without the need for additional hyperparameter tuning. For the Decision Tree model, a fixed random state (random_state = 0) was employed to ensure reproducibility, while other parameters such as tree depth and splitting criteria were retained at their default values to prevent overfitting. The Random Forest model was configured with 100 estimators, a maximum tree depth of 10, and a fixed random state (random_state = 0). These values were selected based on iterative testing and sensitivity analysis to achieve an optimal trade-off between model complexity and prediction accuracy.

For Support Vector Regression, a linear kernel was adopted to capture the underlying relationships in the dataset while maintaining model simplicity, and default regularization parameters were used. The AdaBoost model utilized a Decision Tree as the base estimator and was configured with 100 estimators, a learning rate of 0.1, and a squared loss function. Similarly, the Gradient Boosting model was implemented with 100 estimators, a learning rate of 0.1, a maximum depth of 3, and a fixed random state, ensuring a balance between bias and variance. To further enhance model performance, Grid Search Cross-Validation was applied to the AdaBoost model to optimize the number of estimators. The parameter grid explored values of 50, 100, 200, and 350 estimators, and the optimal configuration was selected based on minimizing the Mean Absolute Error (MAE) using 5-fold cross-validation. In addition, a sensitivity analysis was conducted for the Random Forest model by varying the number of estimators in the range of 10 to 350. The corresponding changes in RMSE, MAE, R^2^, and cross-validation scores were evaluated, confirming that 100 estimators provide a stable and efficient configuration. To ensure robustness and reduce the risk of overfitting, all models were evaluated using k-fold cross-validation with k = 10. In this approach, the dataset was divided into ten subsets, and each subset was used once as a validation set while the remaining subsets were used for training. The final model performance was reported as the average across all folds, thereby improving the reliability and generalizability of the results.

## Results and discussions

### Workability of concrete mixes

The CFV test was utilized to evaluate the workability of AAC incorporating varied proportions of WFS and RHA, ranging from 0–30% and 0–20%, respectively, as presented in Fig. 3. Results indicate a general decrease in CFV with increased WFS and RHA content. A peak CFV of 0.92 and a minimum of 0.84 were observed in mixes where WFS partially replaced RSFA, while CFV ranged from 0.874 to 0.81 with increasing RHA substitution for GGBS. This trend suggests that higher percentages of WFS and RHA reduce CFV due to WFS’s unimodal particle size distribution and its greater specific surface area(SSA)^58^. RHA’s diminutive grain size and high SSA^59^ increase water demand, impacting workability. Nonetheless, the reduction in workability remains within acceptable limits for pavement-quality concrete, achieving a slump range of 25–75 mm, or equivalently, a CFV of 0.92–0.86. This performance suggests that such AAC mixes are viable for high-quality pavement applications, including highways and airfield pavements. Comparatively, mixes with RHA show a notable CFV reduction compared to control samples with 0% RHA, likely due to the cellular structure and hydrophilic nature of RHA, which both raise its SSA and water absorption requirements, as previously discussed^59^. Furthermore, as the RHA replacement level and its fineness increase, a gradual decline in workability is observed^60^, underscoring that elevated SSA correlates with enhanced reactivity, leading to diminished flowability^59^. Similar findings have been noted inOPCC where RHA substituted OPC^61^.

### Density of concrete mixes

To determine the density of the hardened AAC test mixtures, various proportions of RHA and WFS were incorporated, as depicted in Fig. 3. The density of AAC mixtures, with WFS partially substituting RSFA, ranged from 2580 to 2499 kg/m^3^, while mixtures with partial replacement of GGBS by RHA showed densities between 2529 and 2467 kg/m^3^. The density of the hardened AAC mixtures is influenced by the individual densities of the aggregate and binder materials used. As lower-density materials replace higher-density ones, the overall density of the concrete decreases^62^. In this study, WFS and RHA demonstrated lower densities than RSFA and GGBS, respectively, leading to a slight reduction in AAC density with increasing proportions of WFS and RHA. Similar observations were reported by other researchers^63,64^, who found that as RHA, with its lower specific gravity and density than the primary binder (GGBS), is added in higher amounts, the composite density of AAC decreases. The results indicate that even with combined replacements of RSFA and GGBS by WFS and RHA, respectively, the density values remain above 2400 kg/m^3^, aligning with the density standards for structural concrete composites. Density is a critical factor in resisting wear, tear, and abrasion—essential properties for highway pavements and their durability. Thus, the measured density values meet the requirements for their intended design applications.

### Saturated water absorption (SWA) and volume of permeable voids (VPV) values of AAC composites

The SWA value in AAC increased from 3.12 to 4.20%, and the VPV increased from 8.03 to 8.92% when WFS content increased from 0 to 30% in mixtures SA-0-0 and SA-30-0, respectively. The addition of WFS leads to greater porosity due to the rough texture and irregular shape of WFS particles, which form interconnected voids with increased surface area, thus providing more sites for water absorption. When RHA content increased from 0 to 20% in mixtures SA-20-0 and SA-20-20, SWA increased from 3.85 to 5.26%, and VPV increased from 8.49 to 9.09%. This marginal rise in SWA and VPV with higher RHA replacement in AAC is attributed to the finer particle size distribution of RHA compared to GGBS, which may result in a greater volume of voids in the concrete mixture as the RHA content increases. RHA, characterized by a high porosity due to its cellular structure, has a larger volume of voids compared to GGBS. When RHA partially replaces GGBS, it introduces micro-voids into the AAC matrix. Furthermore, the slower pozzolanic reaction of RHA, due to its amorphous silica content, delays the formation of strong binding gels, potentially leaving voids and increasing water absorption within the matrix^65^. The SWA and VPV values are closely linked: as VPV rises, SWA also tends to increase. These properties significantly impact the durability of concrete composites; higher VPV can make the concrete more prone to chemical attack, reducing its durability. However, in the presence of both WFS and RHA, the recorded VPV and SWA values are well within the standard limit of 10%, supporting the material’s suitability for durable pavement construction. The complex interplay of these properties influences porosity, durability, and overall performance in concrete applications^66^.

### CS and MOE value of AAC composites

**Fig. 4:**
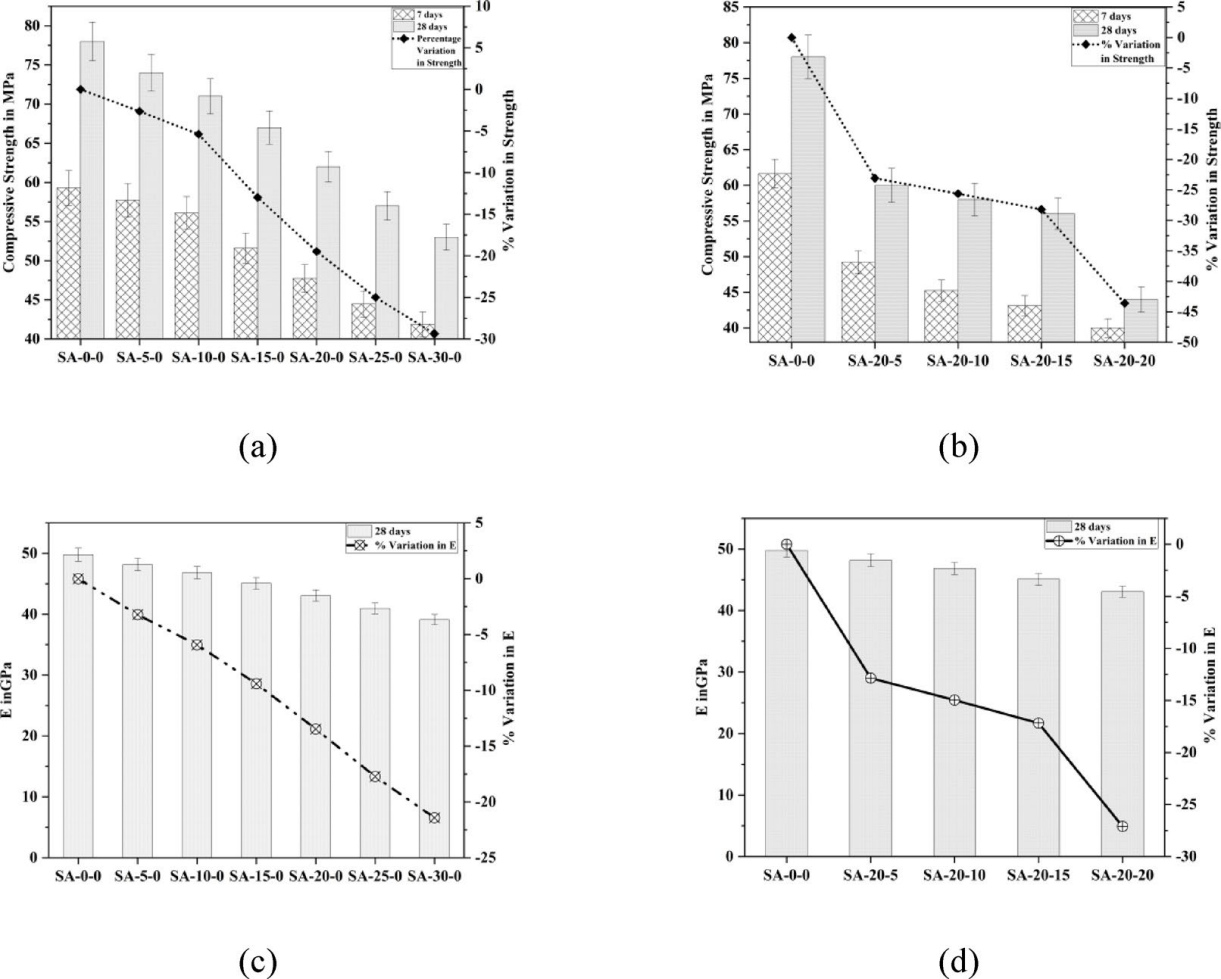
Properties of AAC mixes with varying percentages of WFS and RHA: (**a**) Compressive strength of mixes with different WFS replacement levels; (**b**) Compressive strength of mixes with different RHA replacement levels (with 20%WFS); (**c**) Modulus of elasticity of mixes with different WFS replacement levels; (**d**) Modulus of elasticity of mixes with different RHA replacement levels (with 20%WFS).

The CS and MOE results for AAC mixtures incorporating WFS and RHA are illustrated in Fig. 4. Each mixture’s CS and MOE values were determined by testing 3 specimens, with the final values represented as averages. For AAC containing 0% WFS, the CS reached 62 MPa at seven days, progressing to 78 MPa at 28 days, and achieved an MOE of 49.7 GPa. Increasing WFS replacement levels led to a gradual decrease in CS, with reductions at 28 days of 20.2%, 26.6%, 30.02%, and 35.09% for every additional 5% of WFS replacement up to 30%. Based on CS outcomes, an optimal WFS replacement level was identified at 20%, yielding a CS of 62 MPa that satisfies target requirements. This reduction in strength can be attributed to the increased surface area between WFS and GGBS binder, resulting in weaker interfacial zones as noted in previous studies^66^. The replacement of GGBS with RHA similarly led to a decrease in CS, with the AAC mix containing 0% RHA and 20% WFS reaching 62 MPa at 28 days and an MOE of 43.1 GPa. Incremental RHA replacement of GGBS reduced CS at 28 days by 20.2%, 26.6%, 30.02%, and 35.1% for each additional 5% RHA replacement up to 20%. The optimal RHA substitution level was determined at 15%, achieving a CS of 56 MPa and an MOE of 41.2 GPa, meeting pavement quality concrete requirements. The reference mix SA-0–0 achieves 78 MPa at 28 days, comfortably satisfying the highest-tier PQC requirements for runway-grade and heavy-duty expressway applications. The optimized mix SA-20–15, incorporating 20% WFS and 15% RHA, achieves 56 MPa meeting the M55 PQC specification for national highways and high-traffic urban arterials. This represents a strength reduction of approximately 28% relative to the reference mix but remains within the PQC design range for a large proportion of India’s highway network. Only the extreme combination SA-20-20 the mix referenced when reporting the 35.1% reduction produces a CS (~ 44 MPa) near the M40 minimum threshold, making it suitable only for very low-traffic roads and certainly not for the high-performance pavement categories that motivated this study’s design targets. Importantly, this extreme combination was not selected as the optimized mix; it represents the outer bound of the parametric study. The optimized mix SA-20-15, selected on the basis of the best balance of CS, workability, SWA, and VPV, retains full qualification for national highway-grade PQC applications. While other AAC mixes showed comparable workability and CS values, maximizing the use of waste materials with satisfactory performance was also a consideration. Consequently, the SA-20-15 mix was selected as the optimal AAC mixture. The observed strength reductions may be linked to shifts in hydrate phases within the concrete matrix. GGBS, being more soluble, promotes the formation of dense and robust hydrates, while RHA’s slower dissolution leads to less alumino-silicate gel formation, weakening the matrix. The fine particle size distribution and high surface area of RHA contribute to increased water demand and porosity in the mixture, resulting in weaker interfacial zones and a reduction in both strength and density in the AAC composites. Additionally, higher RHA replacement levels, coupled with an increase in unreactive silica, hindered the polymerization process, further decreasing CS values. The differences in dissolution rates between GGBS and RHA slowed the aluminum sulfate dissolution rate, reducing overall strength^67^. At elevated RHA levels, excess silica may lead to the formation of microcracks, intensifying CS loss in the composite^68,69^. Similar trends were observed in the MOE results, as shown in Fig. 3. Although the incorporation of WFS and RHA resulted in a reduction in compressive strength of up to 35%, the optimized mix (SA-20–15) achieved a 28-day compressive strength of 56 MPa, which satisfies the requirements for pavement-quality concrete as per IRC guidelines. This indicates that the trade-off between sustainability and mechanical performance is acceptable for practical applications^70^. Additionally, the measured density (> 2400 kg/m^3^), and controlled permeability values (SWA and VPV < 10%) indicate adequate durability for field applications.

### Pair plots

The statistical summary of the dataset as represented in Table 5 provides important insights into the distribution and variability of input parameters used for machine learning modeling. The key variables, including GGBS, RHA, RSFA, and WFS, exhibit controlled variability within predefined ranges, reflecting the systematic experimental design adopted in this study^71^. The observed standard deviations indicate moderate variation in these parameters, which is essential for capturing their influence on compressive strength. In contrast, parameters such as NaOH, LSS, water, and coarse aggregate (NCA) remain constant across all samples. While this reduces the overall dimensional variability of the dataset, it enables a focused investigation of the effects of selected material substitutions. As a result, the dataset should be interpreted as representing a constrained design space rather than a fully generalized AAC system. The range and distribution of compressive strength values further indicate that the dataset captures both lower and higher performance mixes, ensuring that the machine learning models are trained across a meaningful spectrum of material behavior. However, the controlled nature of the dataset may influence statistical relationships and should be considered when interpreting model outputs and generalizability.

**Table 5:** Statistical summary of the dataset.

Particulars	count	mean	std	min	25%	50%	75%	max
GGBS	330	470.59	34.04	394.4	443.7	493	493	493
NaOH	330	11.2	0	11.2	11.2	11.2	11.2	11.2
LSS	330	75.15	0	75.15	75.15	75.15	75.15	75.15
W	330	138.02	0	138.02	138.02	138.02	138.02	138.02
NCA	330	1071.4	0	1071.4	1071.4	1071.4	1071.4	1071.4
RHA	330	22.41	34.04	0	0	0	49.3	98.6
RSFA	330	480.66	48.22	404.49	462.27	462.27	520.05	577.84
WFS	330	89.58	44.45	0	53.26	106.53	106.53	159.8
CS	330	61.93	9.88	41	56	60	69	84

GGBS, Ground Granulated Blast Furnace Slag; NaOH, Sodium Hydroxide; LSS, Liquid Sodium Silicate; W, Water; NCA, Natural Coarse Aggregate; RHA, Rice Husk Ash; RSFA, River Sand Fine Aggregate; WFS, Waste Foundry Sand; CS, Compressive Strength.

**Fig. 5:**
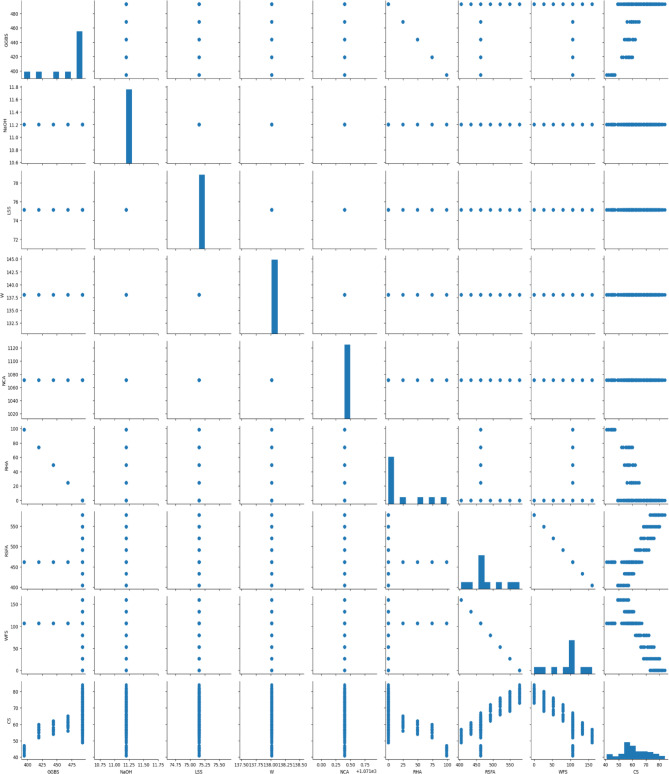
Pair plots for the input parameters and observed responses.

The pair plot presented in Fig. 5 provides a comprehensive visualization of the relationships between input parameters and compressive strength (CS) in alkali-activated concrete (AAC). The diagonal elements represent the distribution of individual variables, while the off-diagonal plots illustrate pairwise interactions, enabling both qualitative and semi-quantitative assessment of trends. A clear inverse relationship is observed between WFS and CS, characterized by a downward trend in the scatter plots, indicating that increasing WFS content significantly reduces compressive strength. Similarly, RHA exhibits a negative and moderately dispersed relationship with CS^72^, suggesting that higher replacement levels lead to strength reduction, although with greater variability compared to WFS. These observations are consistent with the correlation analysis, where WFS and RHA showed strong negative correlations with CS. In contrast, RSFA demonstrates a positive relationship with CS, with data points showing an upward trend, indicating that higher fine aggregate content contributes to improved strength, likely due to enhanced particle packing and reduced voids. GGBS also shows a positive association with CS, although the trend is less pronounced, indicating a moderate influence compared to aggregate-related parameters^73^. The scatter distributions further reveal that variability in WFS and RSFA produces more distinct and structured patterns, highlighting their dominant role in governing compressive strength. Conversely, parameters such as NaOH, LSS, water, and coarse aggregate (NCA) remain constant across the dataset, resulting in no visible trends or correlations with CS. This lack of variability explains their negligible contribution to model predictions and material behavior^74^ Overall, the pair plot analysis confirms that fine aggregate composition (WFS and RSFA) and binder modification through RHA are the primary factors influencing compressive strength, with WFS exerting the most significant negative impact. These findings are consistent with both correlation analysis and feature importance results, reinforcing the reliability of the observed trends^75^.

### Heatmap co-relation

**Fig. 6:**
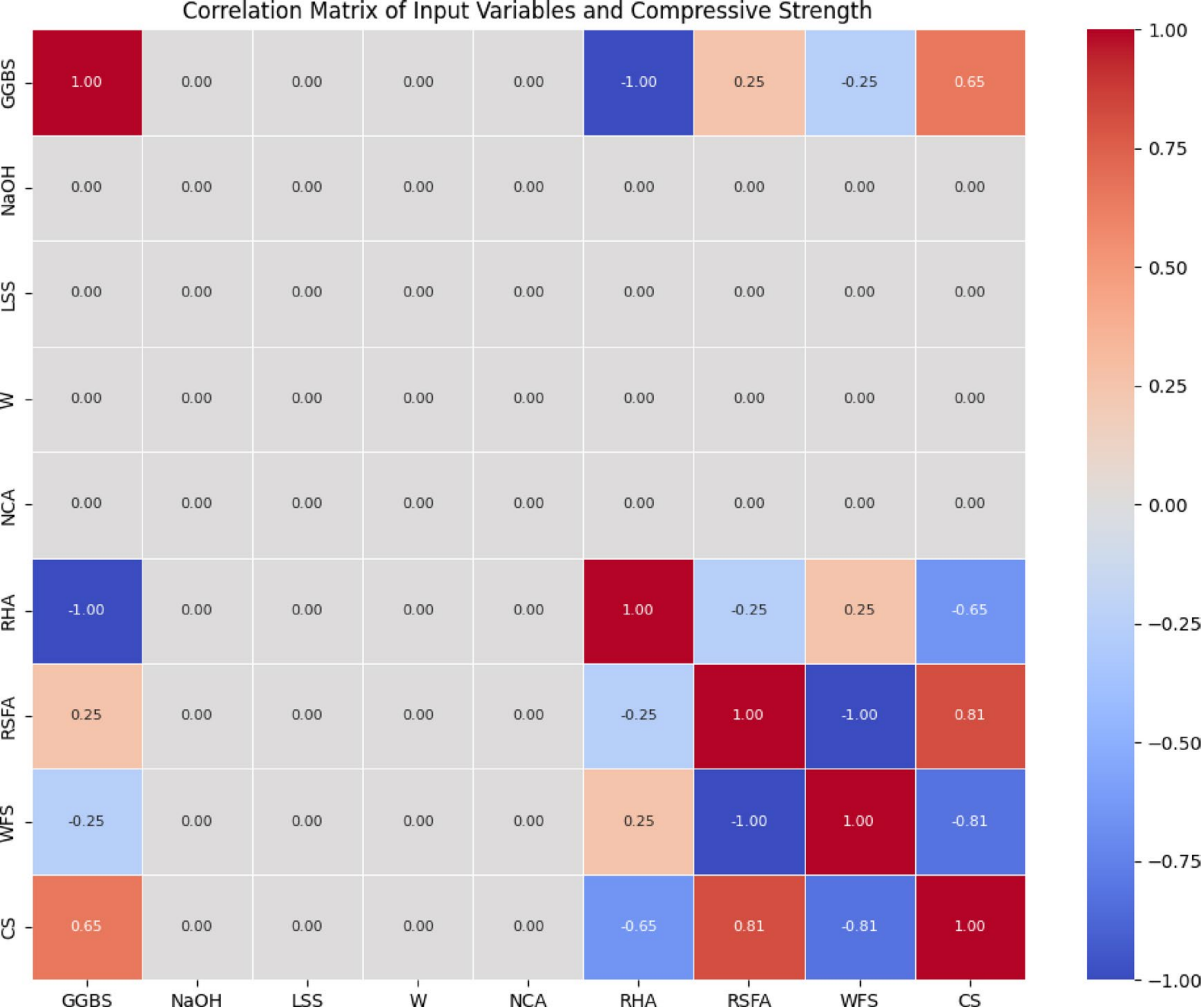
Correlation heatmap of input parameters and observed responses.

The heatmap visualization of correlation coefficients among input parameters and compressive strength (CS), as presented in Fig. 6, provides a quantitative assessment of the relationships governing the AAC system. The correlation coefficients range from − 1 to + 1, where values closer to ± 1 indicate stronger relationships. The results show that RSFA (r = 0.81) and GGBS (r = 0.65) exhibit strong positive correlations with CS, indicating their significant contribution to strength development through improved particle packing and enhanced binder reactivity. In contrast, WFS (r = − 0.81) and RHA (r = − 0.65) demonstrate strong negative correlations with CS, suggesting that higher replacement levels adversely affect strength. This behavior can be attributed to increased porosity, reduced binder density, and lower reactivity at higher substitution levels, which is consistent with previous findings^76,77^. The heatmap also reveals notable interdependencies among input variables, particularly the negative correlations between RSFA and WFS and between GGBS and RHA, reflecting the trade-off inherent in mix design where an increase in one component necessitates a reduction in another. These interactions are critical for optimizing material proportions in AAC systems^78^. Overall, the correlation analysis clearly identifies fine aggregate composition (RSFA–WFS) and binder composition (GGBS–RHA) as the dominant factors influencing compressive strength. These findings are further supported by feature importance analysis, reinforcing the critical role of these parameters in governing AAC performance. While the correlation heatmap provides useful initial insights into the relationships between input variables and compressive strength, the observed correlation coefficients should be interpreted with caution. The dataset is derived from a controlled mix design framework, where parameters such as RSFA and WFS, and GGBS and RHA, are interdependent due to replacement-based proportions. As a result, the relatively high correlation values (e.g., RSFA showing a strong positive correlation with compressive strength) may partly reflect these structural dependencies within the dataset rather than purely independent physical relationships. Therefore, the correlation analysis should be considered as an exploratory tool rather than definitive evidence of causality. To ensure a more robust interpretation, the correlation results are evaluated in conjunction with feature importance analysis and experimental observations, which together provide a more reliable understanding of the governing factors influencing compressive strength.

### Feature importance analysis

To enhance the interpretability of the machine learning models, feature importance analysis was performed using multiple tree-based and ensemble models, including DT, RF, AdaBoost, and GBR. These models inherently provide feature importance scores based on their contribution to reducing prediction error, thereby capturing nonlinear interactions among variables^79^.

**Fig. 7:**
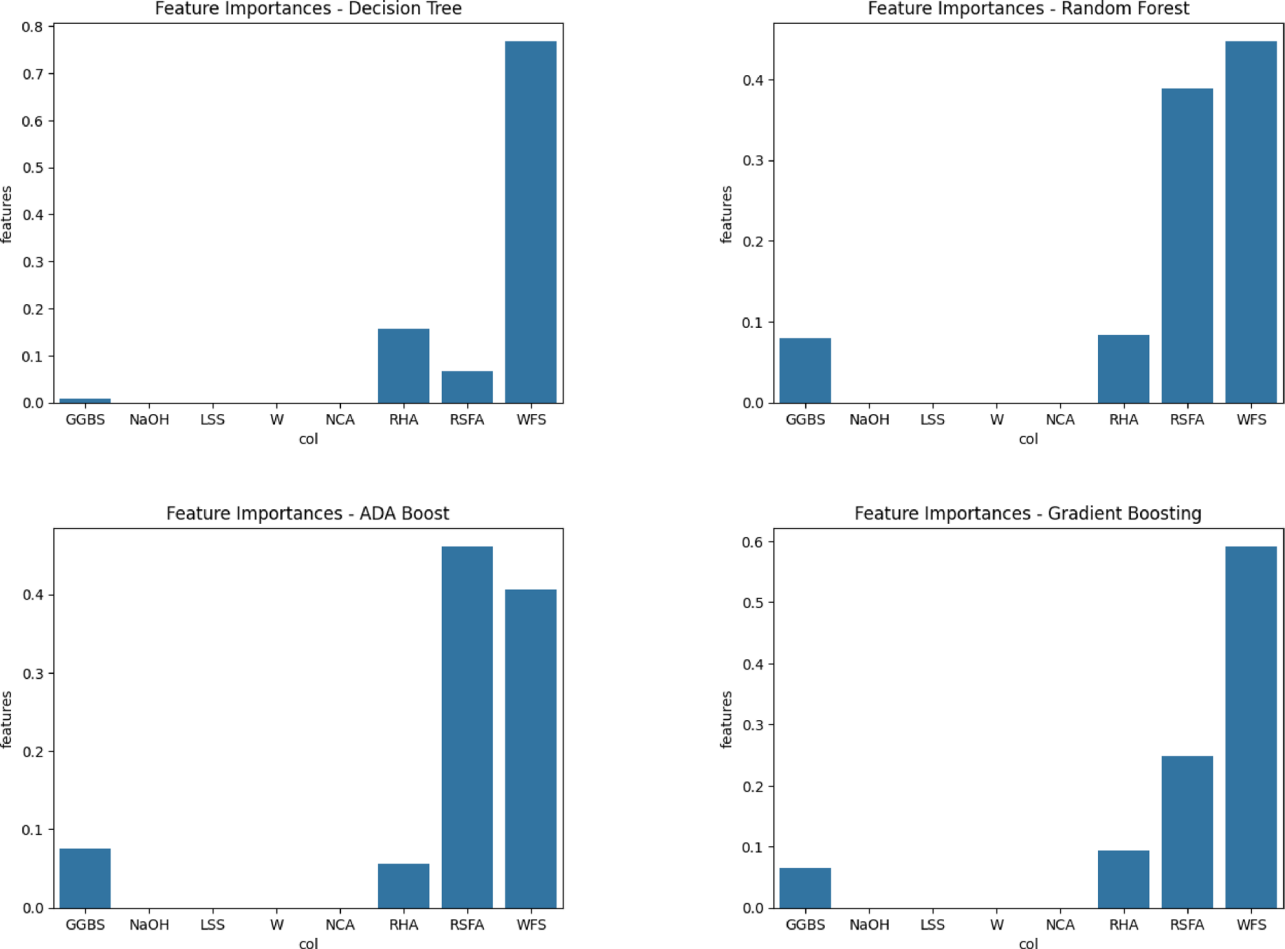
Feature importance analysis of different ML models.

The feature importance results obtained from all models exhibit consistent trends, as shown in Fig. 7. Among all input parameters, WFS is identified as the most influential variable governing compressive strength prediction. This is followed by RSFA and RHA, which show moderate contributions. In contrast, GGBS exhibits relatively lower importance, while parameters such as NaOH, LSS, water, and NCA show negligible influence due to their constant values across the dataset. The dominance of WFS indicates that fine aggregate replacement plays a critical role in determining the mechanical performance of AAC. Higher WFS content significantly affects particle packing, interfacial transition zones, and porosity, thereby influencing compressive strength. Similarly, RHA contributes to strength variation through its impact on binder reactivity and microstructural densification. A comparison across models reveals that ensemble methods such as Random Forest, AdaBoost, and Gradient Boosting produce more stable and consistent importance rankings compared to the Decision Tree model, which shows higher variance and sensitivity to data distribution. The consistency of results across multiple models enhances the robustness and reliability of the findings.

Overall, the feature importance analysis aligns well with experimental observations, where variations in WFS and RHA significantly influenced compressive strength. This agreement between model-based insights and experimental trends strengthens the interpretability of the machine learning framework and provides a deeper understanding of the governing material parameters.

### Relative frequency distribution and statistical and scalar analysis

**Fig. 8:**
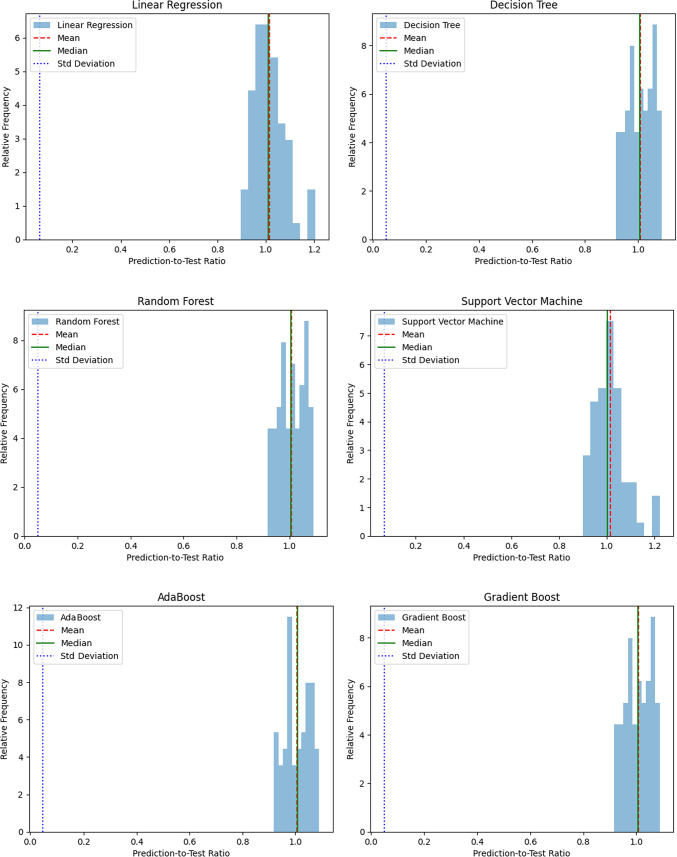
Relative frequency distribution of the prediction-to-test CS ratio.

The analysis of the relative frequency distribution of predicted-to-observed values for various machine learning models, as presented in Fig. 8, provides insight into their predictive behavior and stability. The histograms indicate that all models produce predictions concentrated within a similar range, with most values clustering around unity (ratio ≈ 1.0), suggesting that all models are generally well-calibrated and capable of approximating the observed compressive strength values. The distribution patterns reveal only minor differences among the models^80^. The Decision Tree model exhibits a relatively wider spread and multiple peaks, indicating higher variability and potential sensitivity to data fluctuations^81^. In contrast, ensemble models such as Random Forest, AdaBoost, and Gradient Boosting display comparatively narrower and more uniform distributions, suggesting improved stability. However, the differences in spread and clustering among these models remain relatively small. Statistical indicators further support this observation. The mean and median values for all models are closely aligned around 1.0, indicating minimal bias in predictions. The standard deviation values across models are also low (approximately 0.048–0.067), confirming limited variability. Although slight differences exist the Random Forest (RF), AdaBoost, and Gradient Boosting (GBR) ensembles exhibited superior consistency. Specifically, AdaBoost achieved the lowest standard deviation (0.048), followed closely by RF (0.049), highlighting enhanced stability in predictions compared to the SVR model (0.067), which displayed greater sensitivity to outliers^82^. Similarly, skewness and kurtosis values suggest only minor variations in distribution shape. RF and GBR displayed near-zero, slight negative skewness, indicating a more symmetrical distribution of errors and a lower tendency for systematic overestimation compared to the significant positive skew observed in MLR (1.015) and SVR (0.879)^81^. The negative kurtosis values identified in RF, AdaBoost, and GBR suggest flatter distributions with significantly fewer extreme outlier predictions (“heavy tails”) than the positive kurtosis distributions of MLR and SVR^81^. Overall, the analysis indicates that all models demonstrate comparable predictive capability, with ensemble models showing marginal improvements in stability. These results suggest that the selection of Random Forest as the optimal predictive framework for practical implementation in sustainable PQC mix design.

### Violin diagram for relative error

**Fig. 9:**
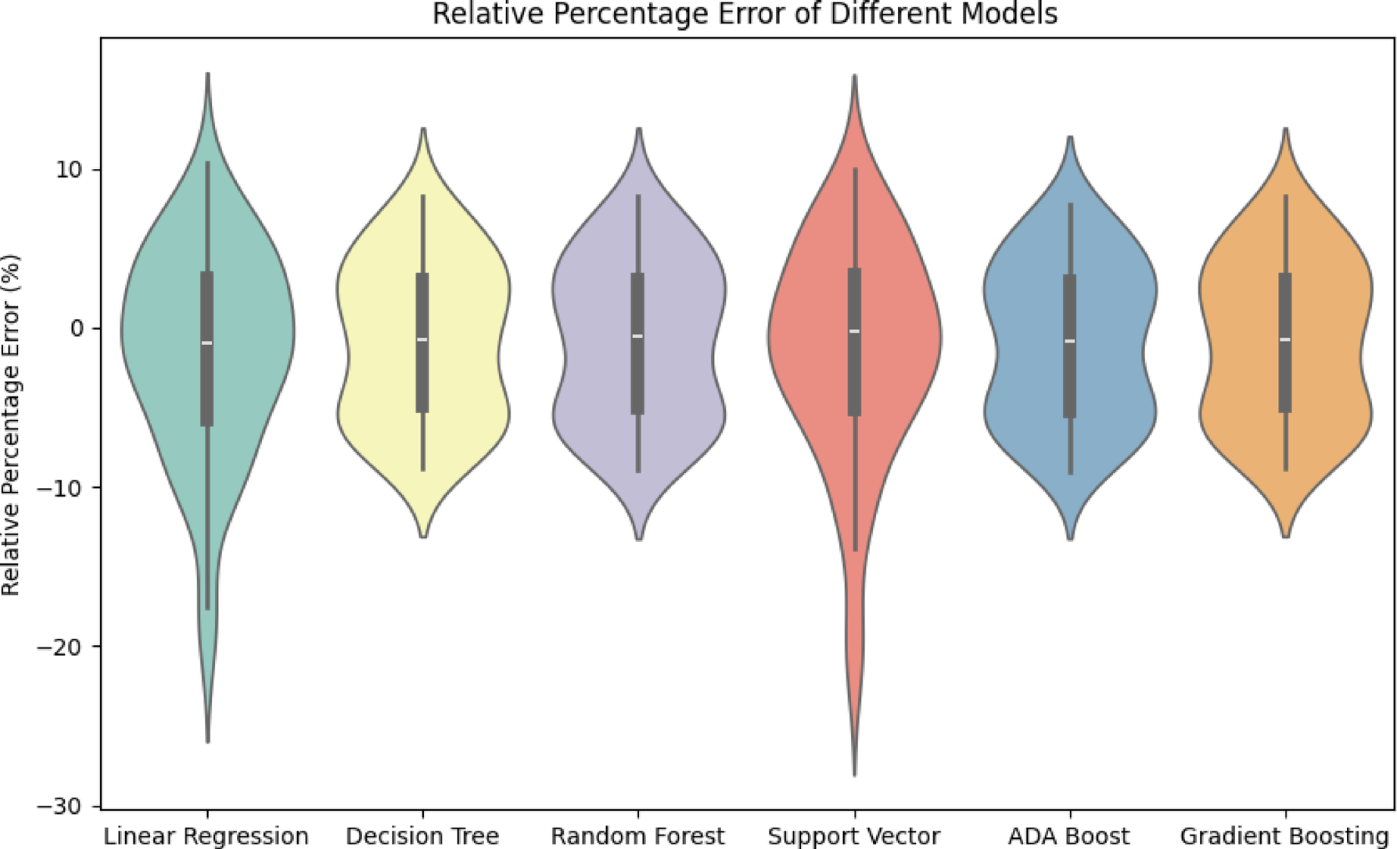
The violin diagram for relative error percentage of different models.

The violin diagram presented in Fig. 9 illustrates the relative percentage error of various predictive models, including MLR, DT, RF, SVR, AdaBoost, and GBR. This visualization effectively conveys the distribution of errors, including their density, central tendencies, and variability, allowing for a comprehensive assessment of model performance. Each violin shape represents the distribution of relative percentage errors for the corresponding model, with the width indicating the density of errors at different levels, facilitating quick visual comparisons. Each violin plot illustrates the distribution of relative percent errors for a given model. The white dot denotes the median error, the thick black bar represents the interquartile range (IQR), and the thin black line indicates the overall data range (excluding outliers). The width of the violin shows the density of errors, enabling visualization of how frequently errors occur at different levels.

Models such as RF and AdaBoost demonstrate narrower IQRs, suggesting greater consistency in their predictions with smaller relative errors. In contrast, the widened tails in the SVR and MLR plots indicate a broader range of outliers, with the SVR frequently producing negative errors, suggesting a tendency for under-predictions. Based on the visualization, RF emerges as the best-performing method due to its lower median relative error and narrow IQR. This conclusion is not drawn from the visualization alone; it is further corroborated by the statistical performance metrics provided in Table 6 (Section “Violin diagram for relative error”), where RF consistently achieved the lowest RMSE, MAE, and MSE, and the highest R^2^ score. Together, the visual and quantitative evidence confirm RF’s superior predictive capability compared to the other models. This finding supports previous research that has identified RF as a superior model for various predictive tasks due to its ability to handle complex datasets effectively^83^. In contrast, models like SVR and MLR present higher error rates and greater discrepancies, indicating less reliable performance compared to their counterparts. The violin diagram effectively conveys the relative error distribution of various models, facilitating a straightforward comparison of their predictive capabilities. With RF exhibiting the most favorable error profile, it is suggested as the optimal choice for applications requiring high accuracy in predictions^84^. The statistical measures accompanying the violin plots further reinforce these observations. The mean and median values for each model provide insights into their central tendencies, while the standard deviations highlight the variability in predictions. Models with lower standard deviations, such as RF and AdaBoost, indicate more consistent performance, while those with higher standard deviations, like SVRandMLR, suggest greater variability and potential for outlier predictions^85^. In summary, the analysis of the violin plots underscores the strengths and weaknesses of various predictive models, with RF emerging as the most reliable option for accurate predictions. This analysis serves as a foundation for selecting appropriate models for future prediction tasks and guides further enhancements in model performance.

**Table 6:** Performance results of the predictive models.

Metrics	MLR	DT	RF	SVR	AdaBoost	GBR
RMSE	3.549605	3.025197	2.986629	3.610154	3.032481	3.025534
MAE	2.953731	2.644507	2.616685	2.930311	2.649002	2.645532
MSE	12.599694	9.151819	8.919952	13.03321	9.195943	9.153854
R^2^ score	0.883626	0.915472	0.917613	0.879622	0.915064	0.915453
CV-mean	0.842492	0.863488	0.86797	0.839946	0.865135	0.863694

RMSE, Root Mean Squared Error; MAE, Mean Absolute Error; MSE, Mean Squared Error; R^2^score, Coefficient of Determination; CV-mean, Cross-Validation Mean.

### Performance results

**Fig. 10:**
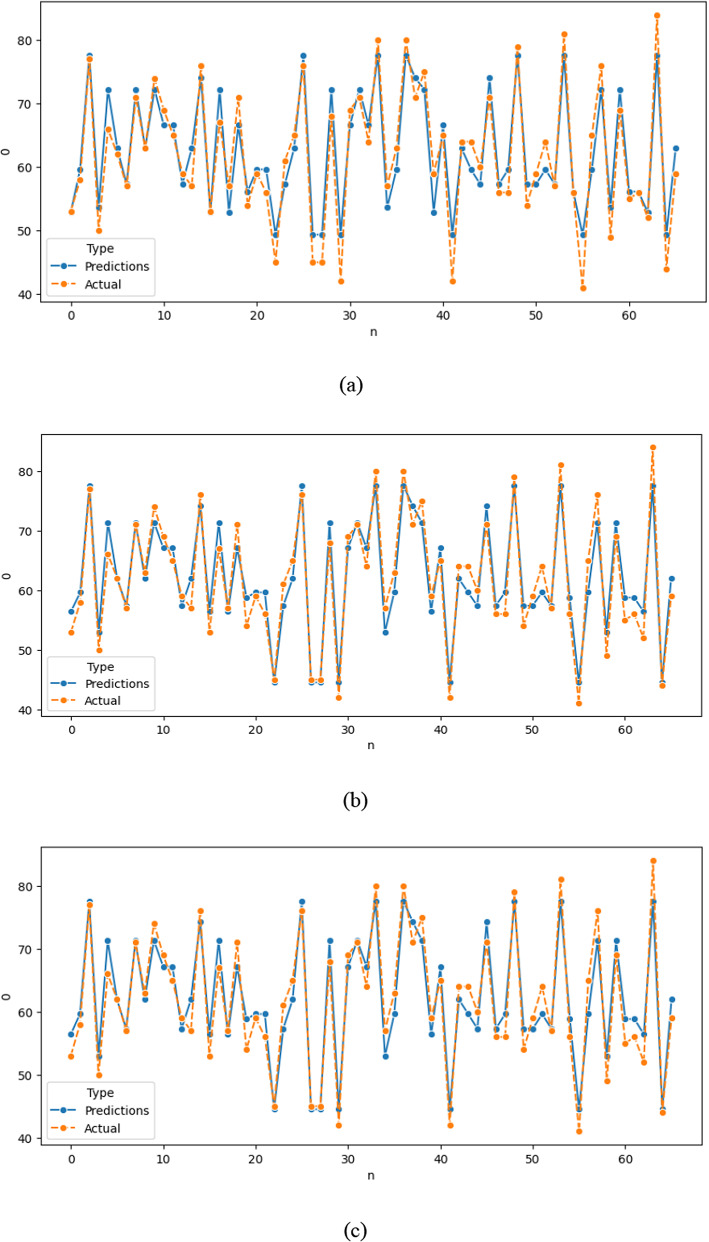

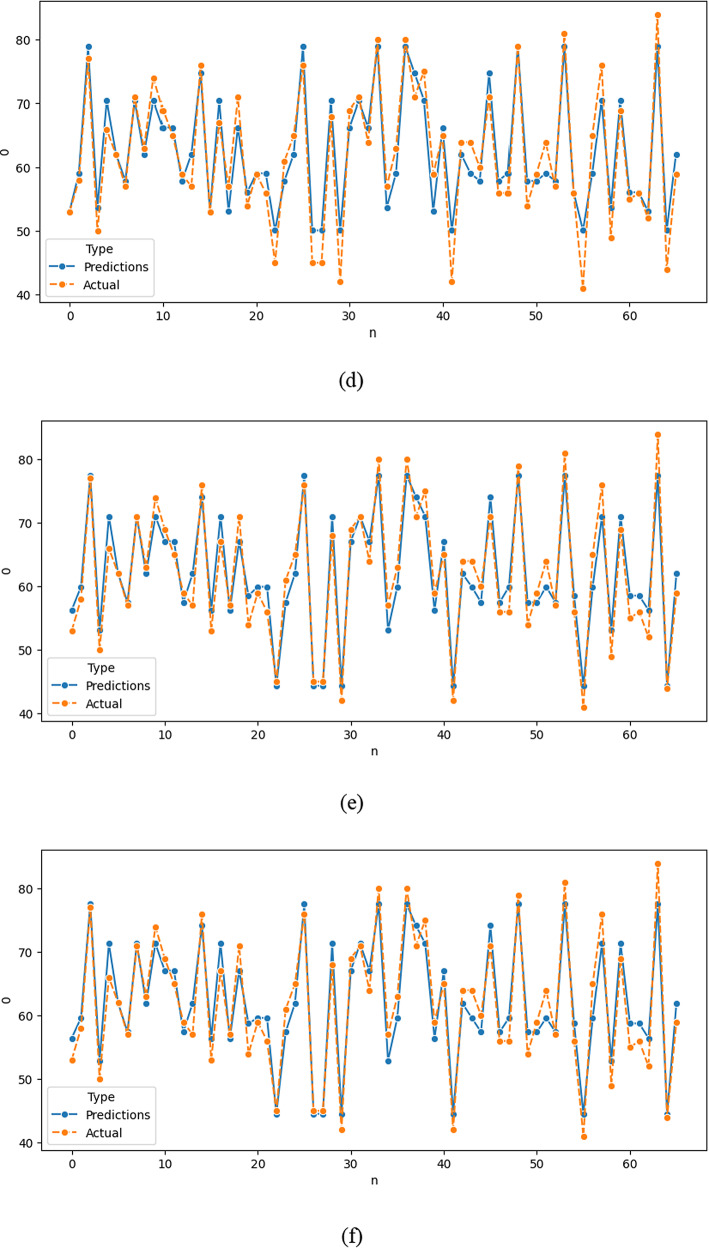
Comparison between actual and predicted values (**a**) MLR (**b**) DT (**c**) RF (**d**) SVR (**e**) ADABoost (**f**) GBR.

The performance results of predictive modelsare effectively illustrated in Fig. 10, where the x-axis (n) represents the index of test samples, while the y-axis (o) indicates compressive strength (i.e., CS in MPa). The orange line with circular markers corresponds to actual CS values, and the blue line with circular markers represents predicted CS values. Their proximity illustrates predictive accuracy, without the use of bar plots. The plots display model performance metrics across cross-validation folds, showing the agreement between predicted and actual CS values for each test sample^86^. Each subplot depicts the accuracy scores represented by blue lines, alongside confidence intervals indicated by orange error bars. RF consistently demonstrates the highest accuracy stability with the least variability across all iterations, maintaining high accuracy levels. On the outset, the plots for DT (Fig. 10b) and RF (Fig. 10c) appear similar at first glance, they reflect distinct model behaviors. The DT model exhibits sharper fluctuations and overfitting tendencies, while the RF reduces such variability by averaging across multiple trees, producing smoother and more reliable predictions. This distinction, though subtle in the visualizations, is confirmed by the performance metrics in Table 6, where RF achieves lower RMSE and higher R^2^ than DT. This observation is supported by literature that emphasizes RF’s robustness and its ability to generalize well across different datasets, making it a preferred choice for various predictive tasks^83^. In contrast, MLR and SVR exhibit fluctuating performance patterns, with accuracy scores dropping considerably in some instances, indicating sensitivity to specific data segments. This aligns with findings that suggest SVR can be sensitive to noise and outliers, which may adversely affect its predictive performance^87^. AdaBoost and GBR show competitive performance, although not as consistently high as RF. Their accuracy tends to fluctuate, especially at the boundaries of the data folds, which is a common characteristic of boosting algorithms that can lead to variability in predictions depending on the data distribution^88^. The DT model displays mixed performance with notable fluctuations in accuracy, suggesting a tendency to overfit to certain data segments, a phenomenon well-documented in DT literature^89^. Based on the performance results, RF is identified as the best method among the models evaluated. Its superior reliability and consistent high accuracy across different iterations make it the most suitable choice for tasks requiring robust predictive performance. It captures the complex, non-linear relationships between input variables and CS of AAC. The ensemble learning approach, which aggregates predictions from multiple decision trees, enhances the model’s robustness and reduces the risk of overfitting. Despite the high accuracy, certain models like SVR showed sensitivity to outliers, which can affect the consistency of predictions, particularly in cases of extreme input values. Additionally, while the models perform well on the given dataset, their accuracy may diminish when applied to significantly different datasets without further calibration. While point metrics such as R2 and RMSE provide an initial measure of model fitness, they do not inherently account for the uncertainty or robustness of predictions across diverse data subsets. To address this, a 10-fold cross-validation (k = 10) was implemented, yielding a CV-mean of 0.8680 for the Random Forest (RF) model. The minimal variance observed across these folds statistically confirms the model’s superior stability and its ability to generalize to unseen alkali-activated concrete (AAC) mix designs. To further quantify prediction uncertainty, parity plots (Fig. 10) were evaluated with ± 10% error bands. The RF model exhibited the highest density of data points within these limits compared to SVR and MLR, which showed significant dispersion and higher sensitivity to outliers. Furthermore, the narrow interquartile ranges (IQR) in the violin diagrams (Fig. 8) demonstrate that the ensemble methods, particularly RF and AdaBoost, produce consistent predictions with low standard deviations (0.049 and 0.048, respectively). These results, supported by negative kurtosis and near-zero skewness, indicate a robust predictive framework that minimizes the risk of extreme outlier predictions in high-performance pavement application.

**Fig. 11:**
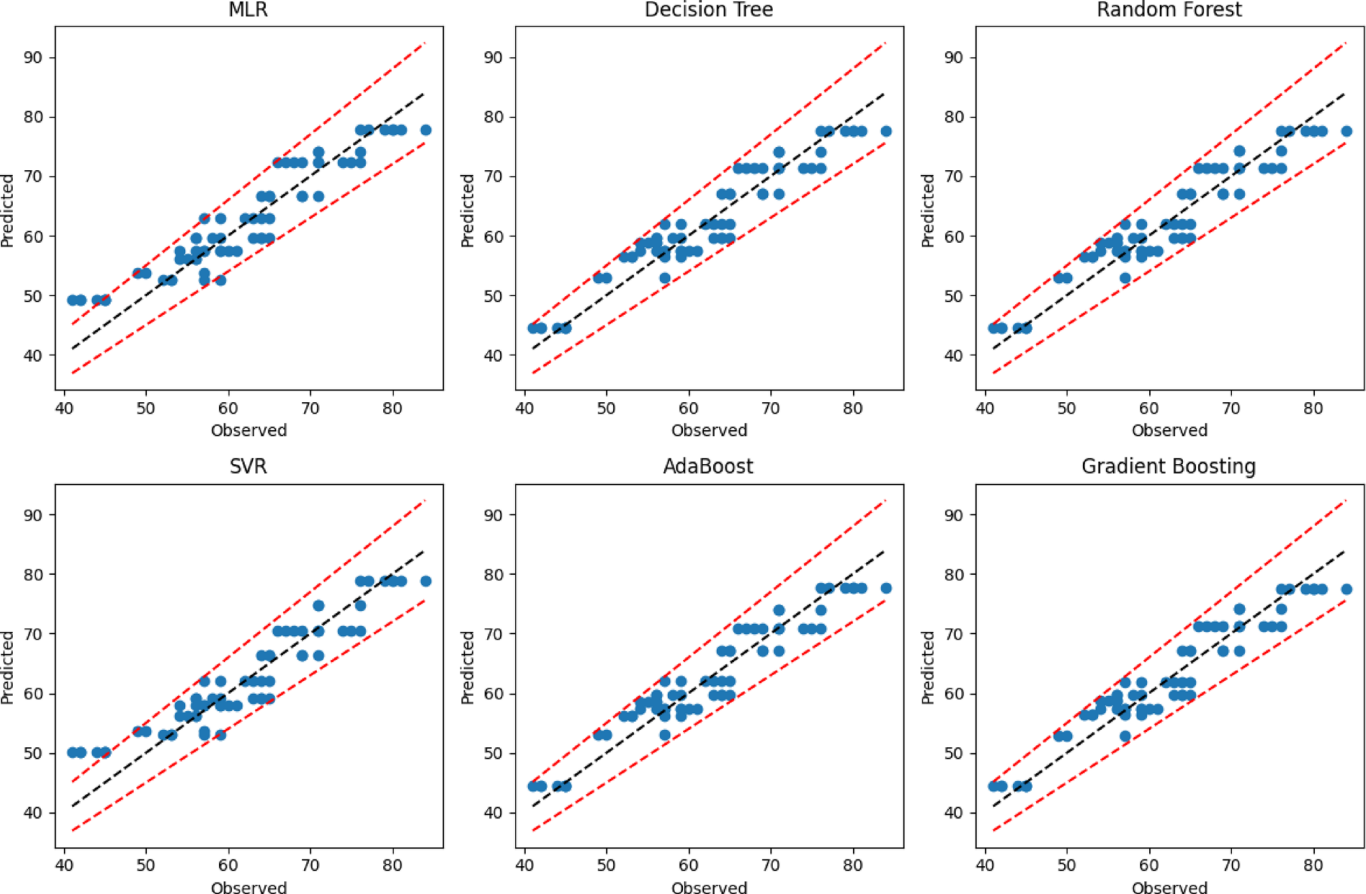
Relationship between predicted and the experimental values of CS of AAC.

Figure 11 illustrates the actual versus predicted values for the predictive models. Each subplot presents a scatter plot with predicted values plotted on the x-axis and actual values on the y-axis, along with a dashed reference line indicating perfect predictions (where predicted equals actual). It is noted that the scatter plots for DT, RF, AdaBoost, and GBR appear visually analogous, with most spots clustering around the reference line, while MLR and SVR show open dispersion. The subtle disparities between these models are more evident in the performance metrics of Table 6, indicating stronger predictive accuracy for RF confirming the relative advantage of RF over the other models.

In the case of MLR, the plot shows a noticeable deviation from the reference line, indicating some underpredictions and overpredictions. The dispersion of points suggests lower accuracy, which aligns with findings that highlight the limitations of linear models in capturing complex relationships within data^80^. The DT plot shows a distribution of points that is somewhat clustered along the reference line, but several outliers indicate that the model may struggle with certain predictions, particularly at the higher end of the range. This is consistent with literature that discusses the tendency of DT to overfit, leading to poor generalization on unseen data^90^. The RF model presents the closest alignment to the reference line, with points tightly clustered around it. This indicates high predictive performance and minimal errors, suggesting it is capable of accurately capturing the underlying data trends. Research has shown that RF is effective in providing robust predictions across various datasets, making it a preferred choice in many applications^91^. The SVR plot shows moderate clustering near the reference line but with several deviations, particularly at the higher and lower extremes, indicating inconsistencies in predictions. This behavior is often attributed to SVR’s sensitivity to the choice of kernel and hyperparameters, which can affect its performance^92^. The AdaBoost scatter plot reveals a relatively strong alignment with the reference line; however, there are notable deviations suggesting some prediction errors, but less so than in other models. This model also shows good alignment with the reference line, resembling RF in terms of performance, though it has slightly more spread in the predictions indicating minor discrepancies. The effectiveness of AdaBoost in improving prediction accuracy through ensemble methods is well-documented^93^. Based on the presented plots, RF emerges as the best-performing method, with its predictions closely following the actual values and exhibiting minimal dispersion. This suggests superior predictive accuracy and robustness compared to the other models evaluated. The findings reinforce the notion that ensemble methods, particularly RF, are advantageous in achieving high accuracy in predictive modeling tasks^94^.

Further, it is imperative to note that Table 6 shows relatively small differences in error metrics across ML models, indicating that all methods provide fair predictive capability for the used dataset. Nevertheless, ensemble approaches (RF, AdaBoost, and GBR) consistently yield lower statistical error margins and superior stability, while MLR and SVRmodels show comparatively higher variability. Thus, while all ML models can be considered satisfactory predictors, RF remains the most trustworthy and reliable choice for practical implementation due to its balance of robustness and statistical accuracy.

Model performance was evaluated using multiple metrics, including the coefficient of determination (R^2^), Root Mean Squared Error (RMSE), and Mean Absolute Error (MAE), to provide a comprehensive assessment of predictive accuracy. While R2 reflects the proportion of variance explained by the model, RMSE and MAE quantify prediction errors in absolute terms, offering complementary insights into model performance. To further assess model robustness and variability, k-fold cross-validation (k = 10) was employed. The cross-validation results indicate consistent performance across different data splits, with relatively low variation in prediction errors, suggesting stable model behavior. MLR exhibits relatively high RMSE (3.5496), MAE (2.9537), and MSE (12.5997), indicating that it may not perform as well on this dataset. An R^2^ score of 0.8836 suggests that MLR explains approximately 88.4% of the variance in the data, which is lower than that of ensemble methods. The cross-validation mean of 0.8425 indicates moderate model reliability. This performance is consistent with findings that suggest MLR model may struggle to capture complex relationships in data, leading to higher error metrics^95^. The DT model shows improved predictive accuracy with RMSE (3.0252), MAE (2.6445), and MSE (9.1518), along with an R^2^ score of 0.9155, indicating it explains approximately 91.5% of the variance. A CV mean of 0.8635 demonstrates reasonable stability. However, while DTs can perform well, they are also prone to overfitting, which can affect their robustness^96^. AdaBoost shows strong predictive capabilities with RMSE (3.0325) and MAE (2.6490) reflecting low error metrics, and an MSE (9.1959) similar to that of the DT. With an R^2^ score of 0.9151, AdaBoost captures a significant portion of data variability. The CV mean of 0.8651 indicates high model reliability and generalizability, which aligns with literature that highlights the effectiveness of AdaBoost in improving prediction accuracy through ensemble methods^95^. RF demonstrates the lowest RMSE (2.9866), MAE (2.6167), and MSE (8.9200), showcasing the highest accuracy in predictions. Its R^2^ score of 0.9176 is the highest among all models, indicating that it captures 91.8% of the data variance. The CV mean of 0.8680 reflects robust reliability and generalizability. RF performance is consistent with research that emphasizes its effectiveness in providing accurate predictions while reducing overfitting compared to simpler models like DTs^97^. SVR exhibits relatively high RMSE (3.6102), MAE (2.9303), and MSE (13.0332), indicating underperformance relative to other models. With an R^2^ of 0.8796, it explains about 87.9% of the variance, the lowest among all models. A CV mean of 0.8399 supports the lower reliability of this model, which is often attributed to its sensitivity to data scaling and inability to capture complex patterns effectively . GBR achieves RMSE (3.0255), MAE (2.6455), and MSE (9.1539), which are close to those of RF and DT, indicating high predictive accuracy. With an R^2^ score of 0.9155, GBR explains 91.5% of the variance, similar to Adaboostand DT. The CV mean of 0.8637 suggests strong generalizability, supporting the notion that GBR is effective in capturing data complexity while providing both accuracy and reliability^98^. Based on these performance metrics, RF emerges as the best model, consistently achieving the lowest error rates (RMSE, MAE, MSE) and the highest R^2^ score (0.9176), indicating superior predictive accuracy and stability. The ensemble-based approach of Random Forest allowed for robust prediction of compressive strength by capturing complex, non-linear interactions among variables. GBR and AdaBoost also exhibited strong predictive capabilities, further validating the utility of ensemble models in optimizing AAC mix designs. AdaBoost and GBR also perform well, offering a balance of accuracy and robustness, making them suitable alternatives. However, due to its lowest error metrics and highest R^2^, RF is recommended as the optimal model for this prediction task. Future analyses could explore further optimization within the AdaBoost framework to refine its predictive capabilities^99^. In conclusion, the performance metrics indicate that RF is identified as a suitable model due to its minimal error metrics and highest R^2^ score, reflecting robust predictive performance and reliability in capturing underlying trends. While AdaBoost and GBR also exhibit strong results, RF’s performance across metrics underscores its potential as the most suitable model for this dataset. Although explicit confidence intervals were not computed in the present study, the combination of multiple evaluation metrics and cross-validation provides a reasonable assessment of model reliability. Future work will focus on incorporating uncertainty quantification approaches, such as prediction intervals and probabilistic modeling, to further enhance the rigor of the analysis).

### Effect of number of estimators

**Fig. 12:**
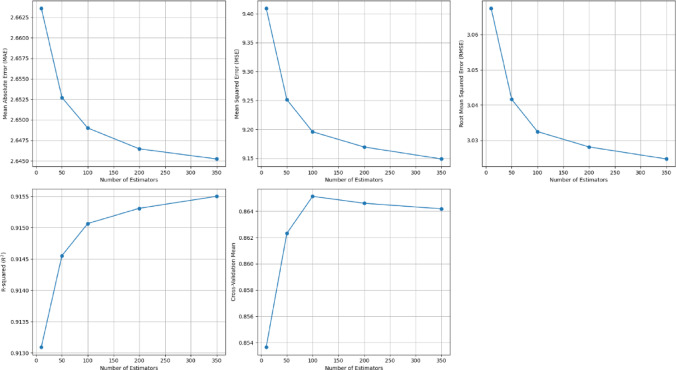
Effect of number of estimators on RF’s performance in terms of (**a**) MAE, (**b**) MSE and (**c**) RMSE (**d**) R^2^ and (**e**) Cross Validation Mean.

**Fig. 13:**
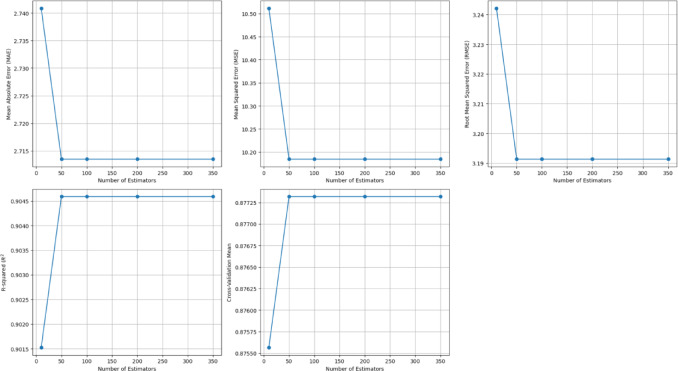
Effect of number of estimators on Adaboost’s performance in terms of (**a**) MAE, (**b**) MSE and (**c**) RMSE (**d**) R^2^ and (**e**) Cross Validation Mean.

**Fig. 14:**
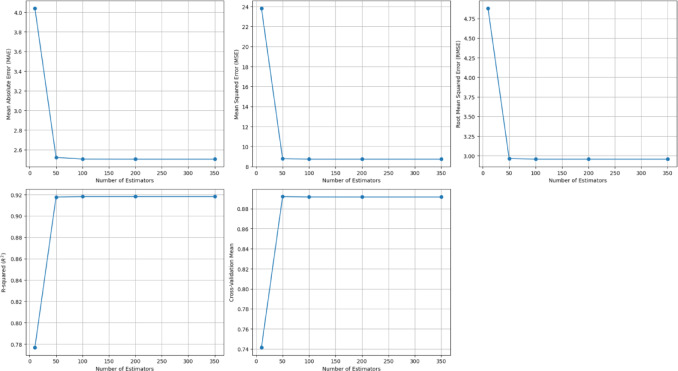
Effect of number of estimators on GBR’s performance in terms of (**a**) MAE, (**b**) MSE and (**c**) RMSE (**d**) R^2^ and (**e**) Cross Validation Mean.

Figures 12, 13, and 14 examines the impact of varying the number of estimators in the Prediction model across several performance metrics. The analysis reveals that MAE and MSE show a significant decline with increasing numbers of estimators, particularly noticeable up to around 150 estimators. Beyond this point, improvements in error reduction are marginal, indicating the law of diminishing returns. This observation is consistent with findings in the literature, which highlight that while increasing the number of trees in a RF generally improves performance, the benefits plateau after a certain threshold^100^. RMSE follows a similar trend, with substantial reductions observed initially, stabilizing as the number of estimators approaches 200–300. Research has shown that the RMSE decreases with the addition of trees, but the rate of improvement diminishes after reaching an optimal number of estimators^91^. The R^2^ metric exhibits a gradual increase with the addition of more estimators, converging toward an asymptotic value around 0.917. This point indicates that the model’s explanatory power stabilizes with further increase in the number of estimators, corroborating studies that emphasize the importance of selecting an appropriate number of trees for optimal model performance^101^. RF models benefit from the ensemble approach, which enhances predictive accuracy and stability as more trees are added. Based on the analysis, it is recommended to use approximately 150 to 200 estimators for optimal performance. Within this range, the model achieves a balanced trade-off between accuracy and computational efficiency, as further increase yield diminishing returns on performance metrics. This suggests a robust predictive capability without unnecessary resource expenditure. The results underscore the importance of selecting an appropriate number of estimators in the RF model, as while more estimators generally enhance model performance, there is an optimal threshold beyond which benefits plateau^102^. In conclusion, adopting around 150–200 estimators it is advised for achieving high accuracy and efficient computational performance in practical applications. Future studies may explore the effects of additional hyperparameter tuning within this framework to optimize performance further^97^.

Figure 13 examines the impact of varying the number of estimators on the performance metrics of the AdaBoost prediction model. The analysis reveals that the MAE shows a dramatic reduction with the increase in the number of estimators, stabilizing at around 50 estimators. Beyond this point, the values plateau, indicating minimal further improvement in prediction accuracy. This finding aligns with existing literature that emphasizes the importance of selecting an optimal number of estimators in boosting algorithms, where performance gains diminish after a certain threshold^103^. Similar to MAE, the MSE experiences a steep decrease initially, particularly in the range of 0 to 50 estimators, and stabilizes afterward. This trend illustrates that a higher number of estimators contributes to diminishing returns in error reduction, which is consistent with research indicating that while increasing the number of estimators can improve model performance, the benefits tend to plateau^93,104^. The RMSE exhibits a comparable trend to both MAE and MSE, showing substantial improvement until approximately 50 estimators, after which it remains constant. This suggests robust predictive performance within this range, reinforcing the notion that careful tuning of the number of estimators is crucial for optimizing AdaBoost’s effectiveness^105^. The R^2^ value increases with the number of estimators, demonstrating improved model fit, and stabilizes around 50 estimators. This indicates that the model captures the variance effectively without significant overfitting, which is a common challenge in ensemble methods. The coefficient of determination follows a similar trend, reflecting an increase in model reliability and predictive capability as more estimators are added, but also stabilizing at the same point. Based on the analysis of the performance metrics, the optimal number of estimators for the AdaBoost model is approximately 50. At this point, the model achieves a balance between minimizing error and maximizing explanatory power, while further increases in estimators yield negligible improvements. The findings confirm the importance of selecting an appropriate number of estimators in the AdaBoost model, as this optimal range not only enhances predictive accuracy but also ensures computational efficiency^106,107^.

Figure 14 illustrates the impact of varying the number of estimators on the performance metrics of the GBR prediction model. The analysis shows that the MAE decreases sharply with an increasing number of estimators, particularly noticeable from 0 to around 50 estimators. This indicates significant improvement in predictive accuracy initially, stabilizing thereafter. Similar trends have been reported in the literature, where the performance of GBR improves with the addition of estimators, particularly in the early stages^108^. The MSE follows a similar pattern, showing a rapid decline up to approximately 50 estimators. Beyond this point, additional estimators contribute to minimal reductions in error, suggesting a point of diminishing returns’ exhibits a comparable trend to both MAE and MSE, showing substantial improvement until approximately 50 estimators, after which it remains constant. This suggests robust predictive performance within this range, reinforcing the notion that careful tuning of the number of estimators is crucial for optimizing GBR’s effectiveness^109^. The R^2^ value demonstrates a consistent upward trend until it stabilizes closer to 50 estimators, indicating that the model’s explanatory power enhances significantly in this range. This metric exhibits stable performance, mirroring the behavior of R^2^, which reinforces the model’s effectiveness in capturing the variance in the dataset^110^. Based on the performance metrics, the optimal number of estimators for the GBR model is approximately 50. At this point, the model achieves a balance between minimizing error rates and maximizing explanatory power. Beyond this threshold, further increase in the number of estimators does not yield substantial improvements, indicating an efficient use of computational resources. The findings underscore the critical role of selecting an appropriate number of estimators in the GBR model, as this optimal range not only enhances predictive accuracy but also ensures computational efficiency^90^. In conclusion, the analysis illustrates that while more estimators generally enhance model performance, there is an optimal threshold beyond which benefits plateau. Adopting around 50 estimators is advised for achieving high accuracy and efficient computational performance in practical applications. While the current dataset has provided substantial insights, we acknowledge that a larger dataset could further enhance model accuracy. The ensemble methods, particularly RF and GBR, have been employed to mitigate potential limitations by leveraging multiple decision trees to reduce overfitting and improve prediction reliability.

## Limitations of the study

The present study does not include fatigue, abrasion resistance, or long-term durability tests, which are critical for evaluating field performance of rigid pavements. Future studies should incorporate these parameters to validate the practical applicability of AAC mixes. In addition, while the use of industrial by-products such as GGBS, RHA, and WFS suggests potential sustainability benefits, a quantitative life cycle assessment (LCA) was not performed. Therefore, the sustainability implications are discussed qualitatively based on material substitution The dataset is characterized by limited variability in certain input parameters (NaOH, LSS, water, and NCA), which may influence the robustness and generalizability of machine learning models. Furthermore, the results presented in this study are based on controlled laboratory conditions. Field implementation may involve additional factors such as environmental exposure, curing variability, and construction practices, which could influence performance. Therefore, further validation through field trials and cost–benefit analysis is necessary to establish large-scale applicability. The dataset size (330 samples) is moderate and derived from controlled experimental conditions, which may not fully represent field variability. These limitations highlight the need for future studies incorporating larger datasets, advanced durability testing, and microstructural analysis to improve the reliability and applicability of the findings. Although ensemble models demonstrated stable and consistent performance, their predictive capability is dependent on the range and distribution of the training data. Therefore, extrapolation beyond the studied parameter space should be approached with caution. Future research should focus on expanding the dataset to include a broader range of input variables, incorporating multi-condition experimental data, and performing external validation to improve model robustness. The integration of uncertainty quantification techniques and advanced feature engineering methods would further enhance the reliability and applicability of machine learning models in AAC design. Future research should focus on integrating external validation and LCA to provide a more comprehensive assessment of both predictive reliability and environmental performance, thereby facilitating practical adoption in pavement applications.

## Conclusion

This study investigates the feasibility of incorporating waste foundry sand (WFS) and rice husk ash (RHA) in air-cured alkali-activated concrete (AAC) for pavement applications, along with the development of machine learning (ML) models for compressive strength prediction. The research establishes a performance-based framework combining sustainable material utilization with data-driven modeling to support pavement-quality concrete (PQC) design.

- The compressive strength (CS) of AAC decreased with increasing replacement levels of WFS and RHA, reaching a maximum reduction of 35.1% at 30% WFS and 20% RHA. However, the optimized mix (SA-20–15) limited the reduction to 30.2% while still satisfying PQC strength requirements, demonstrating that moderate replacement levels can balance sustainability and structural performance.
- Ensemble ML models showed strong predictive capability, with Random Forest (RF), Gradient Boosting (GBR), and AdaBoost exhibiting comparable performance. Although RF achieved the lowest error metrics (RMSE = 2.98, MAE = 2.61), the differences among models were marginal, indicating that multiple ensemble approaches are equally reliable for AAC strength prediction.
- The coefficient of determination values (R^2^ ≈ 0.91–0.92) across ensemble models confirm robust prediction capability; however, the differences are not statistically significant, suggesting that model selection should consider interpretability and stability rather than marginal accuracy gains.
- Feature importance and correlation analysis indicate that GGBS and RSFA are dominant parameters influencing CS, while WFS and RHA govern strength variation through their replacement levels. These relationships should be interpreted within the constrained experimental framework rather than as universal trends.
- Model optimization revealed that 150–200 estimators for RF and approximately 50 estimators for AdaBoost and GBR provided optimal predictive performance, highlighting the importance of tuning ensemble parameters to capture nonlinear material behavior effectively.
- The developed ML models demonstrate strong predictive capability within the defined dataset; however, they should be interpreted as conditional models, applicable within the specified mix design range due to limited variability in certain input parameters.

The findings demonstrate that AAC incorporating WFS and RHA can achieve performance levels suitable for pavement applications while promoting sustainable material utilization. The integration of machine learning provides an efficient tool for mix design optimization, reducing experimental effort and enabling data-driven decision-making.

However, several limitations must be acknowledged. The models are validated only through internal methods and may require retraining for different material sources or environmental conditions. The dataset is constrained by limited variability in certain parameters, and optimization is primarily strength-based without incorporating durability, workability, or long-term performance criteria.

Future research should focus on expanding the dataset with broader input variability, conducting external validation using independent datasets, and incorporating multi-objective optimization frameworks that include durability, workability, and sustainability metrics. Integration of microstructural analysis, long-term performance evaluation, and field trials will be essential for translating these findings into practical pavement applications. Advanced approaches such as transfer learning and hybrid AI techniques can further enhance model generalizability and efficiency.

## Data Availability

The datasets used and/or analysed during the current study available from the corresponding author on reasonable request.
